# Role of Endoplasmic Reticulum Stress in Learning and Memory Impairment and Alzheimer's Disease-Like Neuropathology in the PS19 and APP^Swe^ Mouse Models of Tauopathy and Amyloidosis

**DOI:** 10.1523/ENEURO.0025-17.2017

**Published:** 2017-07-14

**Authors:** Denise Isabelle Briggs, Erwin Defensor, Pooneh Memar Ardestani, Bitna Yi, Michelle Halpain, Guy Seabrook, Mehrdad Shamloo

**Affiliations:** 1 Department of Neurosurgery, Stanford University School of Medicine, Palo Alto, CA 94304-5593; 2 California Innovation Center, Johnson & Johnson, Menlo Park, CA 94025-5232

**Keywords:** amyloidosis, cognition, ER stress, integrated stress response, neurodegeneration, tauopathy

## Abstract

Emerging evidence suggests that endoplasmic reticulum (ER) stress may be involved in the pathogenesis of Alzheimer’s disease (AD). Recently, pharmacological modulation of the eukaryotic translation initiation factor-2 (eIF2α) pathway was achieved using an integrated stress response inhibitor (ISRIB). While members of this signaling cascade have been suggested as potential therapeutic targets for neurodegeneration, the biological significance of this pathway has not been comprehensively assessed in animal models of AD. The present study investigated the ER stress pathway and its long-term modulation utilizing *in vitro* and *in vivo* experimental models of tauopathy (MAPT P301S)PS19 and amyloidosis (APP^Swe^). We report that thapsigargin induces activating transcription factor-4 (ATF4) in primary cortical neurons (PCNs) derived from rat and APP^Swe^ nontransgenic (nTg) and transgenic (Tg) mice. ISRIB mitigated the induction of ATF4 in PCNs generated from wild-type (WT) but not APP^Swe^ mice despite partially restoring thapsigargin-induced translational repression in nTg PCNs. *In vivo*, C57BL/6J and PS19 mice received prolonged, once-daily administration of ISRIB. While the compound was well tolerated by PS19 and C57BL/6J mice, APP^Swe^ mice treated per this schedule displayed significant mortality. Thus, the dose was reduced and administered only on behavioral test days. ISRIB did not improve learning and memory function in APP^Swe^ Tg mice. While ISRIB did not reduce tau-related neuropathology in PS19 Tg mice, no evidence of ER stress-related dysfunction was observed in either of these Tg models. Taken together, the significance of ER stress and the relevance of these models to the etiology of AD require further investigation.

## Significance Statement

Accumulating evidence suggests that endoplasmic reticulum (ER) stress is involved in cellular processes relevant to neuronal survival and death in disorders of the CNS. We assessed the ER stress pathway and the effects of its modulation using *in vitro* and *in vivo* experimental models of tauopathy and excessive amyloidosis. Use of an integrated stress response inhibitor (ISRIB) was not effective at improving the behavioral impairments and neuropathology observed in these models. While no evidence of ER stress or ER stress-related dysfunction involving activating transcription factor-4 (ATF4) and C/EBP-homologous protein (CHOP) was found in these transgenic (Tg) mice, ISRIB partially restored thapsigargin-induced translational repression *in vitro* in primary mouse cortical neurons. In summary, the contribution of ER stress to the etiology of Alzheimer’s disease (AD) warrants further investigation.

## Introduction

Alzheimer’s disease (AD) is a progressive, neurodegenerative disorder characterized by memory loss and global cognitive decline (Alzheimer’s Association, 2013). The neuropathological hallmarks of AD include neuronal loss (Terry et al., 1991) accumulation of extracellular Aβ plaques, and neurofibrillary tangles composed of intracellular aggregates of tau protein (Selkoe, 2001; Schoonenboom et al., 2004; Sobów et al., 2004; Iqbal et al., 2005). Over 46 million people worldwide are currently living with AD or some form of dementia (Prince et al., 2015). This number is expected to exceed 130 million by the year 2050 (Prince et al., 2015). Presently, all approved treatments for AD are geared toward symptom management and do not target the underlying neuropathology. Despite the pressing need for more targeted treatments, to date, all Phase III clinical trials testing therapeutics directed at the neuropathological substrates of AD have failed (Mullane and Williams, 2013; Gauthier et al., 2016). This has intensified the investigation of alternative therapeutic targets implicated in the pathogenesis of AD.

Emerging evidence suggests that endoplasmic reticulum (ER) stress may play an integral role in the development of AD (Paschen and Mengesdorf, 2005a,b; Lindholm et al., 2006; Hoozemans et al., 2009; Scheper and Hoozemans, 2009). A fundamental role of the ER is to ensure that newly synthesized proteins are folded correctly. An aberrant accumulation of unfolded proteins activates multiple signaling pathways collectively referred to as the unfolded protein response (UPR; Spatara and Robinson, 2010). Markers of the UPR have been detected postmortem in the brain tissue of AD patients (Hoozemans et al., 2005; Scheper and Hoozemans, 2015) and UPR activation has been correlated with tau phosphorylation, a critical step preceding the formation of neurofibrillary tangles (Hoozemans et al., 2009). The protein kinase R-like ER kinase (PERK), along with inositol-requiring protein 1, and activating transcription factor-6 (ATF6), are three classes of sensors that recognize unfolded proteins in the ER (Schröder and Kaufman, 2005). In response to ER stress, PERK becomes activated via dimerization and autophosphorylation (Harding et al., 1999; Marciniak et al., 2006) and the collective response of these pathways is referred to as the integrated stress response (ISR; Wek et al., 2006; Sidrauski et al., 2013). On activation, PERK phosphorylates the α-subunit of eukaryotic translation initiation factor-2 (eIF2α; Harding et al., 2000) allowing it to complex with and de-activate elongation initiation factor 2B (eIF2B). With few exceptions, this inhibits global protein synthesis and can alleviate ER stress by preventing further accumulation of unfolded proteins. One exception is the stress-related mRNA ATF4, whose translational efficiency is upregulated by phosphorylation of eIF2α (Harding et al., 2000; Ameri and Harris, 2008). While ATF4 induction can promote the synthesis of pro-survival ER chaperone proteins (Li et al., 2008), it is also a potent inducer of C/EBP-homologous protein (CHOP), a pro-apoptotic transcription factor whose expression is increased under severe or persistent ER stress (Marciniak et al., 2004; Lenna and Trojanowska, 2012).

Previous studies identified a small molecule integrated stress response inhibitor (ISRIB) that targeted selective components of the ER stress pathway and may afford a safer and more tolerable means of target engagement than direct PERK inhibition. ISRIB was reported to improve learning and memory performance in healthy, wild-type (WT) rodents (Sidrauski et al., 2013). *In vitro*, ISRIB mitigated the induction of ATF4 in HEK293 cells challenged with the ER stress inducers thapsigargin and tunicamycin (Sidrauski et al., 2013). Recently, the mechanism by which ISRIB exerts its modulatory control was identified (Sidrauski et al., 2015a). EIF2B dimerizes in response to ER stress, and ISRIB was found to bind and stabilize activated eIF2B dimers, thereby diminishing their sensitivity to eIF2 phosphorylation (Sidrauski et al., 2015b) and lifting the inhibition of protein translation resulting from phosphorylation of eIF2α (Sekine et al., 2015; Sidrauski et al., 2015a,b).

While modulation of eIF2 phosphorylation using ISRIB was found to abate the effects of ER stress *in vitro*, few studies have investigated if ER stress-related dysfunction could be targeted to improve AD-like outcomes *in vivo*. To our knowledge, this is the first study to investigate the role of ER stress and the effects of long-term ISR-related pharmacological modulation on AD-like neuropathology and behavior both *in vitro* and *in vivo*. Specifically, the studies described herein assessed the ER stress pathway and the effects of ISRIB treatment *in vitro* using primary cortical neurons (PCNs) and *in vivo* using experimental AD models of tauopathy (MAPT P301S)PS19 (PS19) and excessive amyloidosis (APP^Swe^).

## Materials and Methods

### Primary neuronal culture

PCNs derived from embryonic day 17 (E17) Sprague Dawley rats (Charles River Laboratories), E18 APP^Swe^ transgenic (Tg) and nontransgenic (nTg) mice (Taconic1349, Tg2567), and C57Bl/6N mice (Charles Rivers Laboratories) were dissociated by incubating cortical tissue in 2 ml of Hibernate E medium (BrainBits) without calcium containing 4 mg papain (Worthington Biochemical) at 37°C for 30 min. Further dissociation was accomplished by trituration using a fire-polished Pasteur pipette (Thermo Fisher). The supernatant was centrifuged (CL2, Thermo Fisher) at 1100 rpm for 1 min and the cell pellet suspended in 2 ml of serum-free MB Activ1l medium (BrainBits) supplemented with an antibiotic solution of penicillin (100 U/ml; Invitrogen) and streptomycin (100 mg/ml; Invitrogen). Cells were counted and seeded onto poly-D-Lysine (Sigma-Aldrich) coated plates. Cells were plated at a density of 1.0 × 10^6^ or 1.5 × 10^6^ cells per well in six-well plates, and 8 × 10^4^ cells per well in 96-well plates. PCNs isolated from rats were cultured for 12–13 days *in vitro* (DIV), and PCNs isolated from APP^Swe^ mice were cultured for 7, 11, or 13 DIV.

### Chemicals

Thapsigargin and puromycin were obtained from Sigma-Aldrich and the trans-isomer of ISRIB from Selleck Chemicals (SKU S7400). The physical and chemical properties of ISRIB were analyzed using high performance liquid chromatography and nuclear magnetic resonance spectroscopy and found to be consistent with those previously reported (analysis by Selleck Chemicals; http://www.selleckchem.com/products/isrib-trans-isomer.html).

### Target engagement and ER stress *in vitro*

To induce ER stress, PCNs were cultured in six-well plates as described above and incubated for 4 h with 1 μM thapsigargin, 1 μM thapsigargin + 200 μM ISRIB, or dimethyl sulfoxide (DMSO; Sigma-Aldrich), which served as a vehicle control. To evaluate the effects of ISRIB on protein synthesis, the SUnSET technique was used as previously described by Schmidt et al. (2009). PCNs isolated from C57Bl/6N mice were cultured for 7 or 11 DIV and challenged with thapsigargin or thapsigargin + ISRIB as described above. Ten minutes before collection, cells were treated with puromycin (10 μg/ml).

#### Immunoblotting

PCNs were washed with 1× PBS (Thermo Fisher) and lysed in M-PER Tissue Protein Extraction Reagent (Life Technologies) with Mini Complete Protease Inhibitor tablet (Roche). Samples were homogenized on ice using an Ultrasonic Probe Homogenizer (Omni International) and centrifuged at 15,000 × *g* for 10 min at 4°C. The supernatants were collected and stored as the soluble fraction. Protein concentration was determined using the BCA protein assay kit (Pierce). Samples were boiled, loaded (40 μg/well) and resolved by 14% Tris-Glycine SDS-PAGE (Life Technologies) electrophoresis under reducing conditions. The protein was transferred to a polyvinylidene difluoride membrane (Life Technologies) and incubated in blocking buffer (0.05% Tween 20, Sigma-Aldrich; 2% normal goat serum, Sigma-Aldrich; 5% nonfat milk, Bio-Rad); 1× TBS, Promega) for 1 h at room temperature. Primary antibodies directed at ATF4 (1:300, Santa Cruz Biotechnology, sc-200), CHOP (1:300, Santa Cruz Biotechnology), puromycin clone 12D10 (1:10,000; EMD Millipore), tubulin (1:300, Sigma-Aldrich), and glyceraldehyde 3-phosphate dehydrogenase (GAPDH; 1:5000; Sigma-Aldrich) were incubated at 4°C either overnight (ATF4, CHOP, puromycin) or for 30 min (tubulin, GAPDH). The following day, membranes were washed (3 × 5 min) with 0.05% Tween 20 in 1× TBS and incubated for 1 h with the appropriate HRP-conjugated secondary antibody (goat anti-mouse, goat anti-rabbit; 1:5000, Life Technologies). Membranes were incubated briefly in ECL substrate (SuperSignal West Dura, Thermo Fisher) and exposed to film (Konica Minolta) for protein detection. ImageJ software (NIH) was used for densitometry analysis of protein levels and expression levels normalized to internal control.

#### Lactate dehydrogenase (LDH) cytotoxicity assay

The LDH assay was conducted according to the manufacturer's protocol (Thermo Fisher). PCNs isolated from E17 rat embryos were cultured in 96-well plates (Thermo Fisher) as described above. Cells were treated with 10 μM thapsigargin, 10 μM thapsigargin + 200 nM ISRIB, 10 μM thapsigargin + 1 μM ISRIB, DMSO vehicle control, or DMSO vehicle control + lysis buffer (positive control) and incubated for 48 h. The media were collected and each sample transferred to a 96-well plate (50 μl/well). The reaction solution was added and allowed to incubate for 30 min. The stop solution was added, and the absorbance read between 490 and 680 nm using a Flex Station 3 microplate reader (Molecular Devices). Percentage cytotoxicity was calculated by subtracting LDH activity of the negative vehicle control from the LDH activity of thapsigargin-treated samples. This value was then divided by the total LDH activity [(maximum LDH release control activity) – (negative control activity)], and multiplied by 100.

### Animals

All animal procedures were performed in accordance with the Stanford University animal care committee's regulations. The studies described herein were conducted in compliance with all applicable sections of the current version of the Final Rules of the Animal Welfare Act Regulations (9 CFR) and the Guide for the Care and Use of Laboratory Animals, Institute of Laboratory Animal Resources, Commission on Life Sciences, National Research Council, 2010. Animals were housed at a standard temperature (22 ± 1°C), in a reverse-cycle light-controlled environment (lights on from 8:30 P.M. to 8:30 A.M.) with *ad libitum* access to food and water. A summary of the mice used for the present studies is provided in Table 1. A total of 194 male mice were used for this study including three-month-old male C57BL/6J mice (*N* = 20, Jackson Laboratory, stock #0664), five-month-old male C57BL/6J mice (*N* = 12), 8-9-month-old male PS19 mice (*N* = 102, Jackson Laboratories, stock #8169, B6;C3-Tg (Prnp-MAPT*P301S)PS19Vle/J), and five- to six-month-old male APP^Swe^ mice (*N* = 60, Taconic, stock #1349, B6;SJL-Tg(APPSWE)2576Kha). Two separate cohorts of PS19 mice were used for our studies. Each cohort was age matched and subjected to the same treatment regimen and behavioral testing schedule. The results from each cohort were pooled for our statistical analysis. One C57BL/6J mouse + ISRIB developed irritation at the injection site during the ninth week of treatment and was sacrificed. In the combined PS19 cohorts, a total of 3 nTg mice + vehicle died over the course of the behavioral experiments. One mouse died in the third week and two mice died in the fifth week. One nTg mouse + ISRIB died in the fifth week. A total of 8 PS19 Tg mice + vehicle died. One mouse died in the first week, six mice died in the fifth week and one mouse died in the sixth week. A total of 6 PS19 Tg mice + ISRIB died. One mouse died in the second week, one mouse died in the third week, three mice died in the fifth week, and one mouse died in the sixth week. A total of seven APP^Swe^ mice died during the second week of treatment: four nTg mice + ISRIB and three Tg mice + ISRIB.

**Table 1.** Total number, genotype, and age of mice used for the present studies are shown

	C57BL/6J	C57BL/6J	PS19	APP^Swe^
Age (months)	3	5	8-9	5-6
*N*	20	12	102	60

### ISRIB preparation and dosing *in vivo*

ISRIB was delivered via intraperitoneal injection in a vehicle consisting of 5% PEG400 (EMD Millipore) and 5% Tween 20 in 1× PBS. To prepare the test article for dosing, ISRIB was weighed out and placed in a 50 ml conical. Appropriate volumes of PEG400, Tween 20, and water were added followed by sonication on ice using a probe homogenizer. PBS followed by water was added to the solution to achieve a stock concentration of 0.5 mg/ml. The stock was further diluted to achieve concentrations of 0.25 and 0.025 mg/ml. Based on an administration volume of 10 ml/kg, the administrative doses of ISRIB were 5, 2.5, and 0.25 mg/kg, respectively. Dosing solutions were prepared fresh daily, protected from light, and used within 24 h. Body weight and predose activity chamber parameters were used to pseudo-randomize and balance treatment groups before the start of treatment. C57BL6/J and PS19 mice received a single daily injection of either vehicle or ISRIB (5 mg/kg) for nine weeks. Over the course of treatment, PS19 mice underwent behavioral testing. APP^Swe^ Tg and nTg mice received a single daily dose of either vehicle or ISRIB (5 mg/kg) on days 1 through 8. In APP^Swe^ mice, daily administration was stopped due to a significant increase in mortality in all mice treated with ISRIB. For the remainder of the study, the dose was reduced and administered only on behavioral test days as follows: vehicle or ISRIB (2.5 mg/kg) 1 h before testing in the Y-maze, vehicle or ISRIB (0.25 mg/kg) immediately after the last trial of the day in the Morris water maze (MWM), and vehicle or ISRIB (2.5 mg/kg) 1 h before the novel object recognition (NOR) and novel object location (NOL) tests. Previous research found that a single dose of ISRIB enhanced behavioral function in healthy, WT mice (Sidrauski et al., 2013). This research informed our decision to modify our paradigm for the remainder of the study and dose only on behavioral test days at the concentrations described above.

### ISRIB pharmacokinetics and tolerability

C57BL/6J mice approximately three months old received a single intraperitoneal injection of either vehicle or ISRIB (5 mg/kg). Brain and plasma were collected at five different time points following the injection (immediately, 0.5, 2, 4, and 8 h after the injection). All animals were anesthetized using isoflurane (Butler Animal Health Supply) gas and euthanized per Stanford University APLAC Guidelines. Blood was collected by transcardial puncture and transferred to plasma separation tubes containing lithium heparin (Becton Dickenson). Tubes containing blood were centrifuged at 4°C at 15,000 × *g* for 2 min. Plasma was transferred to separate Microfuge tubes and stored at -80°C. Mice were perfused with 1× PBS using a low perfusion flow rate to avoid bursting vessels (1-2 on Variable Flow Minipump by VWR). Mice were decapitated and whole brains extracted, frozen on dry ice, and stored at -80°C. Frozen brain tissues were weighed and two volumes of Milli Q water added. Tissues were homogenized on ice using an ultrasonic probe homogenizer. For spiked standards, 25 µl of ISRIB was added to 25 µl of brain tissue homogenate or plasma. For samples, 25 µl of 50% methanol (Fisher Scientific) was used in place of standards. Next, 150 µl of acetonitrile (Fisher Scientific)/methanol 80:20 (v/v) was added to the mixture, vortexed vigorously for 1 min, and centrifuged at 3000 × *g* for 5 min. The supernatant was diluted with water (1:1) and the concentrations of ISRIB in brain and plasma determined using liquid chromatography tandem mass spectrometry. Liquid chromatography separation was conducted on a C18 column (50 × 2.1 mm, 5 µm; Thermo Fisher) with isocratic elution using a mobile phase composed of water and acetonitrile. Formic acid (0.1%; Fisher Scientific) was added to both aqueous and organic phases. The column temperature was set to 25°C and the injection volume was 10 µl. The mass spectrometer was operated in the positive mode with multiple-reaction monitoring. Multiple-reaction monitoring transition of 451.1 → 266.0 was used as the quantifier and 451.1 → 141.0 was used as the qualifier. Data acquisition and analysis were performed using the Analyst 1.6.1 software (AB SCIEX). To ensure mice could tolerate prolonged once daily treatment, C57BL/6J mice approximately five months old were administered either vehicle or ISRIB (5 mg/kg) once daily for 62 consecutive days. Body weight was recorded weekly and notable observations, if any, were recorded daily. On completion of this preliminary study using WT mice, *in vivo* and *in vitro* experiments using PS19 and APP^Swe^ mice were conducted in parallel.

### Behavioral assessment

**Figure 1.**
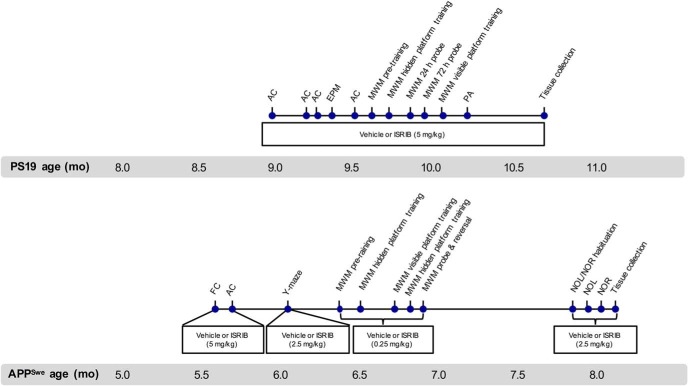
Timeline of behavioral studies conducted in PS19 and APP^Swe^ mice. FC, fear conditioning; AC, activity chamber.

One experienced researcher was assigned to conduct all behavioral tests and remained blinded to the experimental groups throughout the entire in-life phase of the study. Groups were pseudo-randomized using baseline activity chamber performance and body weight as previously stated. Unless otherwise noted, all animals were habituated to the testing area for at least 1 h before testing. Except for the water maze and unless otherwise noted, all apparatuses were cleaned with 1% Vikron solution between subjects. A timeline of the behavioral studies and treatment schedule is provided in Figure 1.

#### Locomotor activity

Locomotor activity was measured in an activity arena (43 cm l × 43 cm w × 30 cm h; Med Associates) equipped with three planes of infrared detectors inside a sound-attenuating chamber (74 cm l × 60 cm w × 60 cm h; Med Associates). Each subject was placed in a corner of the arena and allowed to move freely for 10 min. During that time, activity was recorded using a computer-interfaced infrared beam tracking system (Activity Monitor software V5.93.773; Med Associates). Total activity was defined as the sum of all beam breaks in both the horizontal and vertical planes throughout the entire session. Dependent variables included ambulatory distance, ambulatory duration, ambulatory velocity, time spent in predefined zones, and the number and/or frequency of jumping and rearing. Following the first day of treatment, PS19 mice were tested on days 4, 11, 18, and 25 (Fig. 1) and APP^Swe^ mice were tested on day 7.

#### Anxiety-like behavior

The elevated plus maze (EPM) was used to assess anxiety-like behavior. The EPM apparatus (custom built) consists of two open arms (30 cm l × 5 cm w, with a 0.3 cm h lip around the edges) and two closed arms (30 cm l × 5 cm w × 15 cm h) that extend from a common center (5 × 5 cm). The maze was elevated 63 cm above the floor and the light intensity adjusted to ∼7 lux. Each mouse was gently placed in the center of the maze facing away from the investigator and allowed to move freely for 5 min. Movement was recorded using a WV-CP484 camera (Panasonic) and a computer-interfaced video tracking system (EthoVision XT, version 8.1; Noldus). Dependent variables included the total number of arm entries and time spent in each arm. PS19 mice were tested on day 22.

#### Learning and memory outcomes

##### Fear conditioning

Fear conditioning was used to assess fear-associative learning and recall. Mice were placed in the fear-conditioning chamber (Coulbourne Instruments) for 120 s. After 120 s, a 15-s tone (1700 Hz, 80 dB) was presented. A shock (1.5 mA) was delivered during the last 2 s of the tone. After 120 s, the tone and shock were presented again and 30 s later, mice were removed from the chamber. Contextual and cued retrieval testing occurred 24 h after completion of fear conditioning. To assess context retrieval, mice were placed back in the fear-conditioning chamber for 300 s and the time spent freezing was recorded and analyzed using FreezeFrame software V2.10 (Coulbourne Instruments). Freezing was defined as the complete absence of movement lasting ≥0.75 s. Cued retrieval was assessed 1 h following the completion of contextual retrieval testing. Mice were placed in an unfamiliar context containing different tactile, spatial and olfactory cues for 180 s. After 180 s, mice were presented with a tone (1700 Hz, 80 dB) lasting 180 s, and time spent freezing was recorded. The dependent variable of percentage time spent freezing was used as an index of fear-based learning and memory. APP^Swe^ mice were tested on days 1 and 2.

##### Y-maze

The Y-maze (custom built) was used to assess spontaneous alternation, an exploratory behavior displayed by rodents. The maze is comprised of three arms arranged at 120° angles. Two of the arms are of equal length (15.24 × 12.7 × 7.62 cm) and one arm is longer (20.32 × 12.7 × 7.62 cm). Each mouse was placed in the maze facing away from the center and allowed to move freely for 5 min. Scoring was conducted in real time through live video feed (Roxio adaptor; WV-CP484 camera, Panasonic). Dependent variables including sequence and total number of arm entries were recorded for each mouse. Entry was defined as having all four limbs inside the arm. Alternation was defined as any sequence of three unique arms entries (i.e., “ABC,” “BAC,” but not “CAC”). The percentage alternation rate was calculated as follows: number of alternations/total number of possible alternations*100 and compared with 50% chance alternation. APP^Swe^ mice were tested on day 14.

##### MWM

The MWM was used to assess spatial learning and memory. The maze consists of a round polyethylene tank (172 cm in diameter) filled with water. During training, a platform is submerged 1 cm below the water surface. Tempura paint is added to the water until it becomes opaque and the platform no longer visible. Visual cues are mounted to privacy blinds surrounding the maze. The temperature of the testing room and water was ∼21°C. Movement of the subject was recorded using a computer-interfaced video tracking system (EthoVision XT, version 8.1; Noldus) and the output used for our analysis. Dependent variables included thigmotaxis, swim velocity, latency to locate the platform, and time spent in the target and nontarget zones. The target zone describes the zone that originally housed the platform. The protocol was modified for PS19 and APP^Swe^ mice as described below.

##### MWM protocol for PS19 mice

Pretraining occurred in a single day and consisted of four consecutive trials per mouse. Mice were placed in the maze at the end of a rectangular channel (22 × 172 cm) that led to the platform (22 cm^2^). The drop location alternated between the two short sides of the rectangular channel. Mice were required to locate, climb, and remain atop the platform for 3 s. For hidden platform training, two training sessions were conducted per day for 5 d. Each session consisted of two 90 s trials. The intersession interval was 3–4 h and the intertrial interval was 20–30 min. Pseudo-randomized drop locations were scheduled for each subject in all remaining phases of the test. Each mouse was placed in the maze and allowed to swim freely until they located the platform or the trial ended. Mice that failed to locate the platform were gently guided to its location by the experimenter. Latency to locate the platform was recorded. To assess spatial memory recall, probe tests were conducted 24 and 72 h following completion of hidden platform training. During the probe test, the platform was removed and mice were allowed to swim freely for 90 s. The number of visits to the zone that originally housed the platform (target zone) and time spent in the target and nontarget zones was recorded. Visible platform training occurred in a single day and consisted of four trials. The platform was returned to a new location indicated by a ping-pong ball atop a mast. Each mouse was placed in the maze and allowed to swim freely for the duration of each 90 s trial. Latency to locate the platform was recorded. PS19 mice underwent pretraining on day 28 and hidden platform training on days 29–33. The 24 h probe test was administered on day 34, and the 72 h probe test was administered on day 36. Visible platform training occurred on day 39.

##### MWM protocol for APP^Swe^ mice

General procedures for the MWM were conducted as described above with the following modifications. Pretraining: The channel used in the pretraining phase measured 17 × 172 cm and contained a 17 cm^2^ platform. Hidden and visible platform training: The 17 cm^2^ platform was used on days 1 and 2 of hidden platform training and a 22 cm^2^ platform was used on day 3. Hidden platform training was terminated after 4 d due to learning failures across all groups. Five days later, visible platform training was conducted using the 22 cm^2^ platform and took 3 d to complete. The following day, hidden platform training resumed and lasted for 5 d. Training was conducted in a single session comprised of four trials each day. Each trial was 120 s long separated by an intertrial interval of 35–45 min. The following and final day of testing consisted of a single probe trial followed by four reversal trials. Reversal trials were conducted by moving the platform to a new location. APP^Swe^ mice underwent pretraining on day 28 and hidden platform training on days 29–32. The first round of hidden platform training was terminated because mice failed to acquire the task. APP^Swe^ mice underwent visible platform training on days 41–43. Hidden platform training resumed and occurred on days 44–48. The 24 h probe test was administered on day 49, followed that same day by four reversal trials.

##### Passive avoidance (PA)

The PA test was used to assess fear-based learning and memory. The PA chamber (GEMINI system, San Diego Instruments) consists of a lighted and dark compartment separated by an automated guillotine-style door (gate). Both compartments have a grid floor equipped to deliver electrical shocks. Each mouse was placed into the lighted compartment to habituate to the apparatus. After 30 s, the gate opened allowing access to the dark compartment. As soon as the mouse entered the dark compartment, the gate closed. The following day, mice were placed into the lighted compartment. After 30 s, the gate opened allowing access to the dark compartment. Once the subject crossed into the dark compartment, the gate closed. Following a 3 s delay, a 0.65 mA shock was delivered for 2 s. The following day, mice were again placed into the lighted compartment. After 5 s, the gate opened allowing access to the dark compartment and closed on entry. The dependent variable of latency to enter the dark compartment was recorded and used as an index of fear-based memory. PS19 mice were tested on days 46–48.

##### NOL and NOR

To assess recognition memory, mice were tested using the NOL and NOR. The NOL task assesses the ability of a subject to detect that a familiar object has been moved to a new location. The NOR task assesses the ability of a subject to detect that a familiar object has been replaced with a novel object. Testing occurred in a plastic arena (52 cm w × 52 cm l × 40 cm h) with a white floor, black walls, and a white card fixed to one wall. The placement of the objects as well as the object replaced was pseudo-randomized across subjects. On the first day, mice were habituated to the testing arena and allowed to explore freely for 10 min. The following day, the NOL test was conducted. Mice were placed in the center of the arena containing three identical objects, each placed in a corner, 10 cm from the wall. Mice were then returned to the homecage for 3–4 min. During that time, one of the objects was moved to the previously empty corner. Mice were placed back in the center of the arena and allowed to explore freely for 10 min. Time spent investigating the novel or familiar location was recorded using a computer-interfaced video tracking system (EthoVision XT, version 8.1; Noldus) and the output used for our analysis. For the NOR test, mice were placed in the center of the arena containing three identical objects, each placed in a corner, 10 cm from the wall. Mice were returned to their homecage for 3–4 min. During that time, one of the familiar objects was replaced with an unfamiliar object. Mice were again placed in the center of the arena and allowed to explore freely for 10 min. Time spent investigating the novel or familiar object was recorded. APP^Swe^ mice were tested over days 69–74.

### Exclusions and criteria

Mice exhibiting signs of locomotor impairment, potential blindness, or poor health were excluded from all behavioral tests. During the NOL and NOR tests, mice that traveled <1000 cm during the test trial were excluded from analysis. Mice who met any of the following criteria during the MWM were excluded from our analyses: (1) exhibiting >85% (∼77 s) thigmotaxis in all visible or hidden platform trials; (2) failure to locate the platform in >50% of all visible platform trials; and (3) failure to display learning during hidden platform trials.

### Target engagement *ex vivo*

#### Immunoblotting

On completion of behavioral testing, mice were sacrificed and their brains were removed. The left and right hemispheres were divided sagittally along the midline. One hemisphere was placed in neutral buffered formalin (VWR) for immunohistochemistry (IHC) and the other was frozen on dry ice for analysis by western blot. Frozen brain tissues were homogenized in RIPA buffer (Thermo Fisher) containing protease and phosphatase inhibitor cocktails. Lysates were centrifuged (Sorvall Legend, Thermo Fisher) at 4°C at 10,000 × *g* for 15 min. Supernatants were collected and protein was quantified by the BCA assay. Equal amounts of protein (60, 6, or 10 μg) were resolved by SDS-PAGE and transferred onto a polyvinylidene difluoride (Life Technologies) membrane. Primary antibodies directed against ATF4 (1:300, Santa Cruz Biotechnology, sc-200), CHOP (1:300, Santa Cruz Biotechnology, sc-575), AT8 (1:200, Pierce), Tau-5 (1:200, Abcam), GAPDH (1:5000; Sigma-Aldrich), and tubulin (1:10,000; Sigma-Aldrich) were incubated at 4°C overnight except GAPDH and tubulin, which were incubated for 1 h or 30 min, respectively. Membranes were then washed (3 × 5 min) with 0.05% Tween 20 in 1× PBS, and incubated with the appropriate HRP-conjugated secondary antibody. Signal was detected using SuperSignal West Dura Extended Duration Substrate (Thermo Fisher) and the membranes exposed to film. The integrated density of proteins was quantified using ImageJ software (NIH) and normalized to the appropriate internal control.

#### IHC

Brain tissue was postfixed in formalin for 24 h and transferred to 30% sucrose (Sigma-Aldrich). After saturation in sucrose, brains were frozen in isopentane (Sigma-Aldrich) on dry ice and stored at −80°C. Brains were sectioned coronally at 40 μm and stored at −20°C in cryoprotectant (20% glycerol, 30% ethylene glycol in phosphate buffer). Floating sections were washed in 1× PBS and blocked for 90 min at room temperature in 1× PBS containing 3% bovine serum albumin (Sigma-Aldrich) and 0.3% Triton X-100 (Sigma-Aldrich). Sections were incubated in primary antibody AT8 (1:300, Pierce) in 1× PBS containing 1% bovine serum albumin and 0.1% Triton X-100 at room temperature overnight and then washed with 1× PBS (3 × 15 min). Sections were incubated in red fluorescent Nissl stain for 20 min (1:100, Life Technologies) and/or secondary antibody (488 donkey anti mouse, 1:250, Jackson ImmunoResearch) with DAPI nuclear stain (1:5000; Sigma-Aldrich) for 1.5 h in 1× PBS. Sections were mounted onto slides coated with 0.15% gelatin and coverslipped with polyvinyl alcohol mounting media containing DABCO antifade (Sigma-Aldrich). Images were acquired on a Zeiss Axio Imager 2 microscope. Image based quantifications were performed on two sections per mouse using ImageJ software (NIH). Hippocampal pyramidal cell layer thickness was quantified from Nissl-stained images using the ImageJ line tool. Two sections per animal were imaged at 5× magnification and three measurements per region were taken and used for our analysis. AT8 mean staining intensity and inclusion numbers were quantified using the ImageJ freehand selection and count tool. A reviewer blind to treatment group and genotype using images acquired at 40× magnification performed quantification. Two sections per animal were imaged and the average of two measurements per region were used for our analysis.

### Data analysis

Statistical analysis was performed using GraphPad Prism software (version 6.0b). Statistical tests used for analysis included one-way ANOVA, two-way repeated measures (RM) ANOVA, mixed-measures ANOVA (SPANOVA), paired and unpaired two sample *t* test, one sample *t* test, linear regression, Mantel-Cox, and two-way ANOVA. To correct for multiple comparisons, Bonferroni’s correction was used unless otherwise stated. A summary of the tests used for statistical analysis is provided in Table 2. The average for a particular test point was substituted for missing values due to mortality or exclusion for analyses requiring SPANOVA or two-way RM ANOVA. Outliers were identified using extreme studentized deviate method. Statistics for each outcome measure are described in detail in their respective Results section. Statistical significance was defined at the level of *p* < 0.05. Data are presented as the mean (M) ± standard error of the mean (SEM) unless otherwise noted. For the sake of analyzing phenotype, comparisons were made to nTg vehicle control and the results for PS19 and APP^Swe^ mice are summarized in Tables 3, 4, respectively.

**Table 2.** Summary of the statistical tests used for analyses and corresponding figure(s)

Figure	Statistical test used for analysis
Fig. 2*B*, ATF4 in rat PCNs Fig. 2*D*, LDH in rat PCNs Fig. 3*A*,*C*, ATF4 in APP^Swe^ PCNs Fig. 6*D*, Anxiety-like behavior in PS19 mice Fig. 6*H*, Fear-based learning and memory in PS19 mice Fig. 7*B*, ATF4 in PS19 mice Fig. 8*C*,*D*, P-tau by IHC in PS19 mice Fig. 8*F*, CA1 pyramidal cell layer in PS19 mice Fig. 10*A*,*B*, Locomotor activity in APP^Swe^ mice Fig. 10*D*, Fear-based retrieval in APP^Swe^ mice	One-way ANOVA; Bonferroni’s MCT
Fig. 2*C*, CHOP in rat PCNs Fig. 3*B*, SUnSET Fig. 3*C*, ATF4 in mouse PCNs Fig. 6*F*,*G*, MWM spatial memory recall in PS19 mice Fig. 7*D*, Tau by immunoblot in PS19 mice Fig. 10*F*, MWM spatial memory recall in APP^Swe^ mice Fig. 10*G*, Exploratory behavior in APP^Swe^ mice Fig. 10*H*, Recognition memory in APP^Swe^ mice Tables 3, 4, Phenotype analysis	*t* tests (Student's, one-sample, paired samples)
Fig. 4*A*, PK Fig. 4*B*, Body weight in C57BL/6J mice Fig. 6*A-C*, Locomotor activity in PS19 mice Fig. 6*E*, MWM spatial acquisition in PS19 mice Fig. 5*A*, Body weight in PS19 mice Fig. 9*A*, Body weight in APP^Swe^ mice Fig. 10*C*, Fear conditioning in APP^Swe^ mice Fig. 10*E*, MWM spatial acquisition in APP^Swe^ mice	Two-way ANOVA Linear regression Two-way RM ANOVA or SPANOVA; Bonferroni’s MCT
Fig. 4*C*, Survival rate in C57BL/6J mice Fig. 5*B*, Survival rate in PS19 mice Fig. 9*B*, Survival rate in APP^Swe^ mice	Mantel-Cox

**Table 3.** Summary of the results of the behavioral outcomes used for phenotype analysis in PS19 mice

Behavior	Test	Dependent variable	Genotype	Treatment	Mean ± SEM	*p* value	Interpretation
Locomotion	AC 1	Ambulatory distance (cm)	PS19 nTg	Vehicle	83.64 ± 5.01	—	PS19 Tg mice displaya hyperactive phenotypeevidenced by increasedlocomotor activity
				ISRIB	97.50 ± 6.89	ns	
			PS19 Tg	Vehicle	82.65 ± 6.73	ns	
				ISRIB	105.48 ± 5.87	ns	
	AC 2	Ambulatorydistance (cm)	PS19 nTg	Vehicle	79.88 ± 4.43	—	
				ISRIB	78.79 ± 5.60	ns	
			PS19 Tg	Vehicle	98.85 ± 7.06	ns	
				ISRIB	103.98 ± 5.26	*p* < 0.05	
	AC 3	Ambulatorydistance (cm)	PS19 nTg	Vehicle	75.09 ± 5.16	—	
				ISRIB	70.62 ± 4.98	ns	
			PS19 Tg	Vehicle	90.88 ± 8.72	ns	
				ISRIB	95.82 ± 6.62	*p* < 0.05	
	AC 4	Ambulatorydistance (cm)	PS19 nTg	Vehicle	79.78 ± 5.28	—	
				ISRIB	78.59 ± 5.85	ns	
			PS19 Tg	Vehicle	86.06 ± 10.40	ns	
				ISRIB	92.07 ± 6.42	ns	
Anxiety-likebehavior	EPM	Open arm duration (%)	PS19 nTg	Vehicle	10.67 ± 1.55	—	PS19 Tg miceexhibit reduced anxiety-like behavior
				ISRIB	14.14 ± 2.23	ns	
			PS19 Tg	Vehicle	24.99 ± 3.53	*p* < 0.001	
				ISRIB	22.80 ± 2.91	*p* < 0.01	
Spatial acquisition learning	MWM D1	Escape latency (s)	PS19 nTg	Vehicle	52.26 ± 3.86	—	PS19 Tg mice displayimpairments in spatiallearning with modestrestoration by ISRIB
				ISRIB	60.11 ± 3.52	ns	
			PS19 Tg	Vehicle	83.22 ± 1.94	*p* < 0.0001	
				ISRIB	74.89 ± 6.55	*p* < 0.01	
	MWM D2		PS19 nTg	Vehicle	30.15 ± 3.90	—	
				ISRIB	33.01 ± 3.89	ns	
			PS19 Tg	Vehicle	58.06 ± 6.04	*p* < 0.0001	
				ISRIB	55.05 ± 6.61	*p* < 0.01	
	MWM D3		PS19 nTg	Vehicle	19.38 ± 2.50	—	
				ISRIB	24.03 ± 3.61	ns	
			PS19 Tg	Vehicle	43.58 ± 6.60	*p* < 0.0001	
				ISRIB	37.15 ± 4.50	ns	
	MWM D4		PS19 nTg	Vehicle	18.32 ± 3.21	—	
				ISRIB	22.36 ± 4.50	ns	
			PS19 Tg	Vehicle	43.08 ± 7.72	*p* < 0.001	
				ISRIB	28.38 ± 6.16	ns	
	MWM D5		PS19 nTg	Vehicle	12.56 ± 1.32	—	
				ISRIB	16.87 ± 3.59	ns	
			PS19 Tg	Vehicle	41.98 ± 6.54	*p* < 0.0001	
				ISRIB	26.54 ± 4.04	ns	
Spatial memory recall	Prove test 24 h	Quadrantduration (%)	PS19 nTg	Vehicle	−24.45 ± 2.71	*p* < 0.0001	PS19 Tg mice display impaired spatial memory recall
				ISRIB	−21.54 ± 3.55	*p* < 0.0001	
			PS19 Tg	Vehicle	−6.43 ± 6.89	ns	
				ISRIB	−4.26 ± 5.28	ns	
	Prove test 72 h		PS19 nTg	Vehicle	−20.84 ± 3.07	*p* < 0.0001	
				ISRIB	−15.33 ± 4.20	*p* < 0.01	
			PS19 Tg	Vehicle	2.57 ± 6.76	ns	
				ISRIB	−2.76 ± 5.70	ns	
Fear learningand memory	PA, trainingvs testing	Latency to cross (s)	PS19 nTg	Vehicle	117 ± 22	*p* < 0.0001	PS19 Tg mice do not exhibit deficits in fear-based learning and memory
				ISRIB	137 ± 24	*p* < 0.0001	
			PS19 Tg	Vehicle	155 ± 28	*p* < 0.0001	
				ISRIB	100 ± 22	*p* < 0.001	

Comparisons made to nTg vehicle control. AC, activity chamber; D1-5, day 1-5.

**Table 4.** Summary of the results of the behavioral outcomes used for phenotype analysis in APP^Swe^ mice

Behavior	Test	Dependent variable	Genotype	Treatment	Mean ± SEM	*p* value	Interpretation
Locomotion	AC	Ambulatory distance (cm)	APP^Swe^ nTg	Vehicle	108.20 ± 9.45	—	APP^Swe^ Tg mice exhibit locomotor hyperactivity
				ISRIB	88.75 ± 6.40	ns	
			APP^Swe^ Tg	Vehicle	124.80 ± 9.78	ns	
				ISRIB	139.50 ± 15.10	ns	
		Center duration (s)	APP^Swe^ nTg	Vehicle	90.97 ± 12.30	—	
				ISRIB	83.92 ± 13.13	ns	
			APP^Swe^ Tg	Vehicle	173.40 ± 13.15	*p* < 0.01	
				ISRIB	154.50 ± 21.89	*p* < 0.05	
Fear-associated learning	Baseline	Freezing (%)	APP^Swe^ nTg	Vehicle	0.38 ± 0.23	—	APP^Swe^ Tg mice display impairments in fear-associated learning
				ISRIB	2.82 ± 2.34	ns	
			APP^Swe^ Tg	Vehicle	1.50 ± 1.26	ns	
				ISRIB	0.57 ± 0.37	ns	
	Tone 1		APP^Swe^ nTg	Vehicle	5.69 ± 2.33	—	
				ISRIB	8.43 ± 4.31	ns	
			APP^Swe^ Tg	Vehicle	2.72 ± 1.61	ns	
				ISRIB	1.90 ± 1.39	ns	
	ITI1		APP^Swe^ nTg	Vehicle	22.83 ± 6.21	—	
				ISRIB	24.18 ± 4.67	ns	
			APP^Swe^ Tg	Vehicle	11.62 ± 5.76	ns	
				ISRIB	8.79 ± 3.27	ns	
	Tone 2		APP^Swe^ nTg	Vehicle	30.36 + 8.32	—	
				ISRIB	39.02 ± 8.62	ns	
			APP^Swe^ Tg	Vehicle	17.13 ± 5.71	ns	
				ISRIB	14.15 ± 4.93	ns	
	ITI2		APP^Swe^ nTg	Vehicle	52.64 ± 6.80	—	
				ISRIB	60.00 ± 5.69	ns	
			APP^Swe^ Tg	Vehicle	18.26 ± 3.56	*p* < 0.0001	
				ISRIB	12.42 ± 3.62	*p* < 0.0001	
Fear-based retrieval	Context based		APP^Swe^ nTg	Vehicle	49.45 ± 6.35	—	APPSwe Tg exhibit impaired context-based fear memory retrieval
				ISRIB	50.76 ± 5.95	ns	
			APP^Swe^ Tg	Vehicle	25.93 ± 3.63	*p* < 0.01	
				ISRIB	20.10 ± 4.03	*p* < 0.001	
	Cue based		APP^Swe^ nTg	Vehicle	50.12 ± 10.06	—	APP^Swe^ Tg mice do not display impairments in cue-based fear memory retrieval
				ISRIB	56.82 ± 7.39	ns	
			APP^Swe^ Tg	Vehicle	32.64 ± 5.32	ns	
				ISRIB	34.35 ± 6.74	ns	
Spatial acquisition learning	MWM D1	Escape latency (s)	APP^Swe^ nTg	Vehicle	33.10 ± 4.88	ns	APP^Swe^ Tg do not exhibit impairments in spatial memory acquisition
				ISRIB	16.08 ± 3.15	ns	
			APP^Swe^ Tg	Vehicle	32.56 ± 5.84	ns	
				ISRIB	24.57 ± 6.55	ns	
	MWM D2		APP^Swe^ nTg	Vehicle	31.44 ± 5.30	ns	
				ISRIB	12.66 ± 2.79	ns	
			APP^Swe^ Tg	Vehicle	26.21 ± 5.01	ns	
				ISRIB	29.79 ± 6.50	ns	
	MWM D3		APP^Swe^ nTg	Vehicle	23.02 ± 3.74	ns	
				ISRIB	9.58 ± 1.93	ns	
			APP^Swe^ Tg	Vehicle	19.19 ± 2.90	ns	
				ISRIB	19.92 ± 3.92	ns	
	MWM D4		APP^Swe^ nTg	Vehicle	25.95 ± 4.86	ns	
				ISRIB	6.91 ± 1.21	ns	
			APP^Swe^ Tg	Vehicle	21.12 ± 3.41	ns	
				ISRIB	14.86 ± 1.82	ns	
	MWM D5		APP^Swe^ nTg	Vehicle	16.56 ± 3.15	*p* < 0.01	
				ISRIB	19.04 ± 7.50	ns	
			APP^Swe^ Tg	Vehicle	17.29 ± 2.99	ns	
				ISRIB	12.30 ± 1.49	ns	
	MWM R		APP^Swe^ nTg	Vehicle	66.35 ± 7.55	*p* < 0.0001	
				ISRIB	37.79 ± 9.25	ns	
			APP^Swe^ Tg	Vehicle	71.21 ± 6.16	*p* < 0.0001	
				ISRIB	58.59 ± 8.69	*p* < 0.0001	
Spatial memory recall	Probe test	Duration (%)	APP^Swe^ nTg	Vehicle	−13.65 ± 4.63	*p* < 0.05	APP^Swe^ Tg display intact spatial memory recall
				ISRIB	−27.80 ± 7.17	*p* < 0.05	
			APP^Swe^ Tg	Vehicle	−18.06 ± 5.72	*p* < 0.05	
				ISRIB	−18.74 ± 6.18	*p* < 0.05	
Working memory	Y-maze	Alternation (%)	APP^Swe^ nTg	Vehicle	62.17 ± 1.71	*p* < 0.0001	APP^Swe^ Tg mice display impaired working memory
				ISRIB	56.77 ± 3.52	ns	
			APP^Swe^ Tg	Vehicle	56.23 ± 3.71	ns	
				ISRIB	55.46 ± 3.61	ns	
Recognition memory	NOR	Duration (%)	APP^Swe^ nTg	Vehicle	−28.39 ± 10.47	*p* < 0.05	APP^Swe^ Tg exhibit deficits in recognition memory
				ISRIB	1.95 ± 19.43	ns	
			APP^Swe^ Tg	Vehicle	9.34 ± 13.48	ns	
				ISRIB	7.32 ± 12.88	ns	

Comparisons made to nTg vehicle control. AC, activity chamber; ITI, intertrial interval; D1-5, day 1-5; R, reversal trial.

## Results

### ER stress and target engagement *in vitro*

#### Thapsigargin induces ATF4, CHOP, and cytotoxicity in rat PCNs with partial restoration by ISRIB

**Figure 2.**
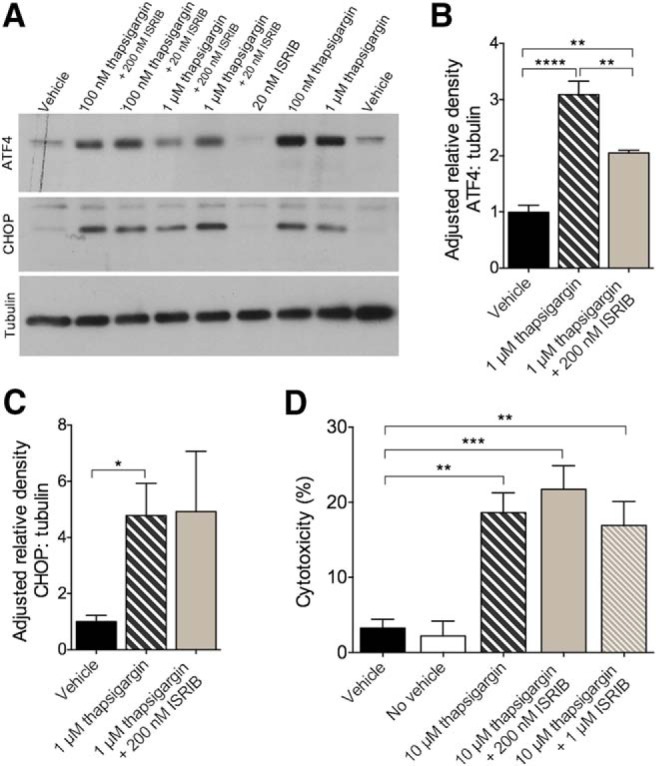
Thapsigargin induced ER stress and target engagement *in vitro*. ***A***–***D***, ER stress-induced ATF4 translation, but not CHOP activation or cytotoxicity, is reduced by ISRIB in rat PCNs. ***A***, Representative immunoblots of primary cortical cell lysates derived from E17 Sprague Dawley rats probed using antibodies directed against ATF4, CHOP, and tubulin. ***B***, Quantification of ATF4 levels normalized to tubulin. ATF4 is increased in cells treated with 1 μM thapsigargin or 1 μM thapsigargin + 200 nM ISRIB compared to vehicle control. Cells treated with 1 μM thapsigargin have more ATF4 compared to cells treated with 1 μM thapsigargin + 200 nM ISRIB. Vehicle, *n* = 6; 1 μM thapsigargin, *n* = 5; 1 μM thapsigargin + 200 nM ISRIB, *n* = 4. ***C***, Quantification of CHOP levels normalized to tubulin. CHOP is increased in cells treated with 1 μM thapsigargin compared to vehicle control. Vehicle, *n* = 4; 1 μM thapsigargin, *n* = 3; 1 μM thapsigargin + 200 nM ISRIB, *n* = 3. ***D***, Quantification of cytotoxicity by LDH. Cells treated with 10 μM thapsigargin, 10 μM thapsigargin + 200 nM ISRIB, or 10 μM thapsigargin + 1 μM ISRIB have higher percentages of cytotoxicity compared to vehicle control. Vehicle, *n* = 6; no vehicle, *n* = 6; 10 μM thapsigargin, *n* = 6; 10 μM thapsigargin + 200 nM ISRIB, *n* = 6; 10 μM thapsigargin + 1 μM ISRIB, *n* = 6. Error bars indicate SEM; *p < 0.05, **p < 0.01, ***p < 0.001, ****p < 0.0001.

Thapsigargin is a potent ER stress inducer (Thomenius and Distelhorst, 2003; Kim et al., 2008) that has been successfully used in immortalized cell lines (Sidrauski et al., 2013). ISRIB was previously found to block PERK-mediated induction of ATF4 in HEK293 cells challenged with thapsigargin and tunicamycin (Sidrauski et al., 2013) and CHOP in U2OS cells challenged with tunicamycin (Sidrauski et al., 2013). To investigate the ER stress pathway *in vitro* and to ensure target engagement, PCNs derived from E17 Sprague Dawley rats were treated with 100 nM or 1 μM thapsigargin. To assess the ability of ISRIB to mitigate these effects, cells were additionally treated with 20 nM or 200 nM ISRIB. While the IC50 of ISRIB-A1 was originally reported at 5 nM (Sidrauski et al., 2013), a recent report cited assay-dependent IC50 values of 27–35 nM (Sekine et al., 2015). Accordingly, we chose to use a minimally effective dose (20 nM) and one that was ∼10-fold above cell-based IC50 values (200 nM). The effects of thapsigargin and ISRIB treatment on the induction of ATF4, CHOP, and cytotoxicity in rat PCNs are shown in Figure 2. The main effect of treatment on levels of ATF4 (one-way ANOVA; *F*_(2,12)_ = 43.91, *p* < 0.0001) was significant (Fig. 2*B*). Compared to cells treated with DMSO (vehicle control), cells treated with 1 μM thapsigargin (Bonferroni’s MCT, *p* < 0.0001) and 1 μM thapsigargin + 200 nM ISRIB (Bonferroni’s MCT, *p* = 0.0024) had higher levels of ATF4. Compared to cells treated with 1 μM thapsigargin, cells treated with 1 μM thapsigargin + 200 nM ISRIB had lower levels of ATF4 (Bonferroni’s MCT, *p* = 0.0038). Compared to vehicle control, cells treated with 1 μM thapsigargin (*t*_(5)_ = 3.805, *p* = 0.0126) had significantly higher levels of CHOP (Fig. 2*C*). Cells treated with 1 μM thapsigargin + 200 nM ISRIB also had higher levels of CHOP compared to cells treated with vehicle, although the results were not significant (*p* = 0.0824). While chronic ER stress is known to compromise cell viability and induce apoptosis (Tabas and Ron, 2011; Verfaillie et al., 2013) the role of the PERK- eIF2α pathway in the regulation of cell death is not entirely clear (Kim et al., 2008). To investigate if any effect of eIF2B-related modulation by ISRIB might be found downstream of CHOP, we challenged rat PCNs with high molarity thapsigargin to induce cytotoxicity. The effects of thapsigargin and thapsigargin + ISRIB on cytotoxicity are shown in Figure 2*D*. Using the LDH assay, the main effect of treatment on cytotoxicity (one-way ANOVA; *F*_(3,20)_ = 9.513, *p* = 0.0004) was significant. Compared to cells treated with vehicle control, cells treated with 10 μM thapsigargin (Bonferroni’s MCT, *p* = 0.0028), 10 μM thapsigargin + 200 nM ISRIB (Bonferroni’s MCT, *p* = 0.0004), or 10 μM thapsigargin + 1 μM ISRIB (Bonferroni’s MCT, *p* = 0.0079) had increased cytotoxicity. No significant differences were observed in cells treated with thapsigargin compared to cells treated with thapsigargin + ISRIB (*p* > 0.9999). These data confirm that thapsigargin is a potent generator of ER stress and that it induces the translation of key modulators of the UPR. Thapsigargin-induced ATF4 translation was mitigated by the addition of ISRIB- further confirming appropriate target engagement. Not surprisingly, ISRIB did not confer neuroprotection against thapsigargin at challenge doses beyond those capable of inducing CHOP. Together, these results indicate that ER stress can be successfully modeled and modulated *in vitro*, using thapsigargin and ISRIB in rat PCNs. Furthermore, the results of this experiment were used to guide our *in vitro* studies in the APP^Swe^ model of amyloidosis.

#### APP^Swe^ PCNs show no evidence of ER stress-related dysfunction in vitro despite evidence of ISRIB target engagement

**Figure 3.**
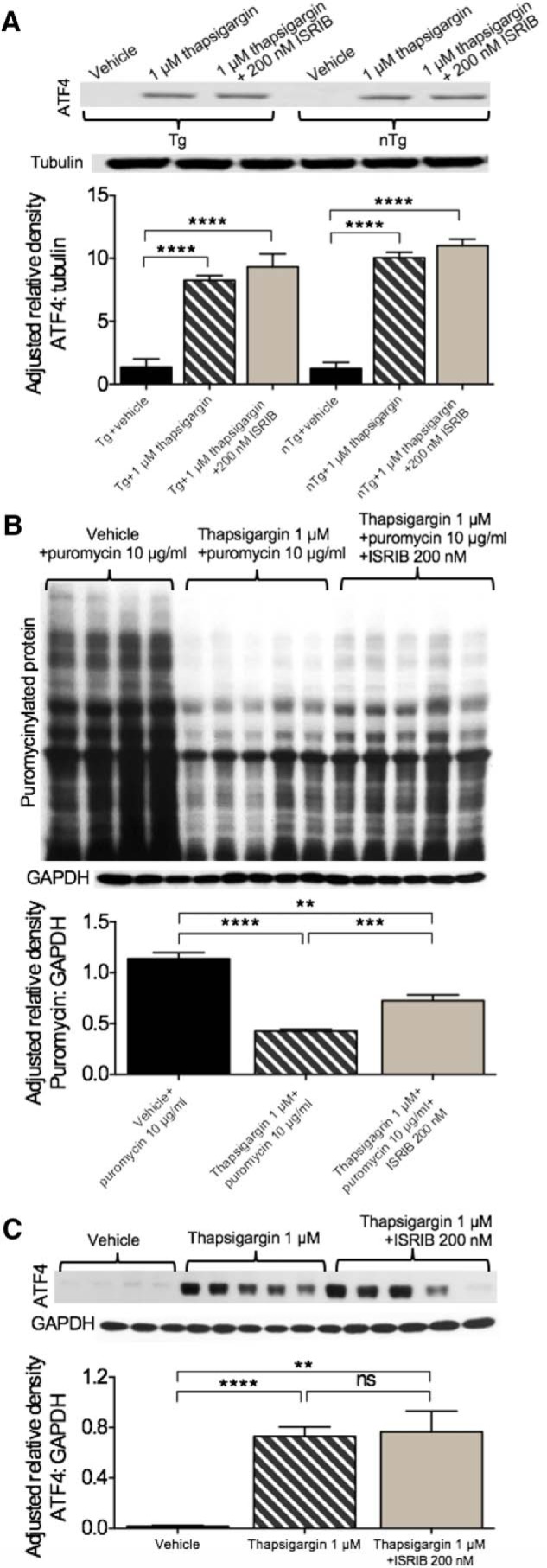
ER stress-related dysfunction is not observed in the APP^Swe^ model *in vitro* despite evidence of ISRIB target engagement. ***A***, Thapsigargin-induced ER stress is not mitigated by ISRIB in APP^Swe^ mouse PCNs. Cells from nTg and Tg mice cultured for 13 DIV treated with 1 μM thapsigargin or 1 μM thapsigargin + 200 nM ISRIB have increased levels of ATF4 compared to vehicle control. nTg vehicle, *n* = 3; nTg + 1 μM thapsigargin, *n* = 3; nTg + 1 μM thapsigargin + 200 nM ISRIB, *n* = 3; Tg vehicle, *n* = 3; Tg + 1 μM thapsigargin, *n* = 3; Tg + 1 μM thapsigargin + 200 nM ISRIB, *n* = 3. ***B***, Thapsigargin (1 μM) attenuates protein synthesis in nTg PCNs cultured for 7 DIV. ISRIB (200 nM) provides partial restoration of protein synthesis in PCNs challenged with thapsigargin. nTg vehicle + 10 μg/ml puromycin, *n* = 4; nTg + 1 μM thapsigargin + 10 μg/ml puromycin, *n* = 5; nTg + 1 μM thapsigargin + 200 nM ISRIB, *n* = 5. ***C***, Thapsigargin-induced ER stress is not mitigated by ISRIB in nTg mouse PCNs. Cells from nTg mice cultured for 7 DIV treated with 1 μM thapsigargin or 1 μM thapsigargin + 200 nM ISRIB have increased levels of ATF4 compared to vehicle control. nTg vehicle, *n* = 6; nTg + 1 μM thapsigargin, *n* = 6; nTg + 1 μM thapsigargin + 200 nM ISRIB, *n* = 6. Error bars indicate SEM; **p < 0.01, *** *p* = 0.001, ****p < 0.0001.

Because ER stress has been implicated in the pathogenesis of AD, we assessed its involvement and response to thapsigargin *in vitro* using PCNs generated from APP^Swe^ nTg and Tg mice. In addition, beta-site APP-cleaving enzyme 1 (BACE1), the enzyme responsible for increased production of Aβ in APP^Swe^ mice, is one of few exceptions whose mRNA translation is upregulated by eIF2α phosphorylation (De Pietri Tonelli et al., 2004; Lammich et al., 2004; Mihailovich et al., 2007; O'Connor et al., 2008; Devi and Ohno, 2010; Scheper and Hoozemans, 2015). To investigate the ER stress pathway and the effects of its modulation, cells were treated with vehicle, 1 μM thapsigargin, or 1 μM thapsigargin + 200 nM ISRIB as guided by our previous experiments. Because thapsigargin successfully induced, and ISRIB successfully mitigated, the induction of ATF4 in rat PCNs, we focused on this marker in APP^Swe^ PCNs (Fig. 3). The main effect of treatment on levels of ATF4 (one-way ANOVA; *F*_(5,12)_ = 50.80, *p* < 0.0001) was significant (Fig. 3*A*). Compared to nTg cells treated with vehicle control, nTg cells treated with 1 μM thapsigargin (Bonferroni’s MCT, *p* < 0.0001) and 1 μM thapsigargin + 200 nM ISRIB (Bonferroni’s MCT, *p* < 0.0001) had increased levels of ATF4. Similarly, Tg cells treated with 1 μM thapsigargin (Bonferroni’s MCT, *p* < 0.0001) and 1 μM thapsigargin + 200 nM ISRIB (Bonferroni’s MCT, *p* < 0.0001) had increased levels of ATF4 compared to cells treated with vehicle. No differences were found between genotypes in levels of ATF4, regardless of treatment. To ensure these outcomes were not the result of inadequate ISRIB target engagement, the experiment was repeated in nTg cells cultured for 7 and 11 DIV. Half of the cells were allocated for analysis using the SUnSET technique (Schmidt et al., 2009) to examine the effects of ISRIB on global protein synthesis. The remaining half were used to examine the effects of ISRIB on thapsigargin-induced ATF4 expression (as described above). The results of the SUnSET experiment in cells cultured for 7 DIV are shown in Figure 3*B*. Cells treated with thapsigargin + puromycin (M = 0.4275, SD = 0.03957) had reduced levels of puromycinylated protein compared to cells treated with vehicle + puromycin (M = 1.139, SD = 0.1167); *t*_(7)_ = 12.93, *p* < 0.0001. Cells treated with thapsigargin + ISRIB + puromycin (M = 0.7267, SD = 0.1228) had reduced levels of puromycinylated protein compared to cells treated with vehicle + puromycin; *t*_(7)_ = 5.115, *p* = 0.0014. Finally, cells treated with thapsigargin + ISRIB + puromycin (M = 0.7267, SD = 0.1228) had increased levels of puromycinylated protein compared to cells treated with thapsigargin + puromycin (M = 0.4275, SD = 0.03957); *t*_(8)_ = 5.185, *p* = 0.0008. This experiment was repeated in cells cultured for 11 DIV and similar results were found (data not shown). In this experiment, cells treated with thapsigargin + puromycin and thapsigargin + ISRIB + puromycin had reduced levels of puromycinylated protein compared to cells treated with vehicle + puromycin (*p* < 0.0001 and *p* = 0.001, respectively). Again, cells treated with thapsigargin + ISRIB + puromycin had increased levels of puromycinylated protein (*p* = 0.0013) Compared to cells treated with thapsigargin + puromycin. Having established proof of ISRIB target engagement, as evidenced by restoration of protein synthesis, we next evaluated the levels of ATF4 in response to thapsigargin challenge in cells cultured for 7 DIV (Fig. 3*C*). Cells treated with thapsigargin (M = 0.7311, SD = 0.1841) had increased levels of ATF4 compared with cells treated with vehicle (M = 0.01943, SD = 0.01355); *t*_(10)_ = 9.444, *p* < 0.0001, as did cells treated with thapsigargin + ISRIB (M = 0.7651, SD = 0.4040); *t*_(10)_ = 4.518, *p* = 0.0011. This experiment was repeated in cells cultured for 11 DIV (data not shown) and similar results were found. Cells treated with thapsigargin or thapsigargin + ISRIB had increased levels of ATF4 compared to cells treated with vehicle (*p* < 0.0001 and *p* = 0.0122, respectively). While cells cultured for 11 DIV treated with thapsigargin + ISRIB had a modest reduction in levels of ATF4 compared to cells treated with thapsigargin-only, the results were not significant (*p* = 0.137). Together, these results indicate that PCNs cultured from APP^Swe^ mice showed no evidence of ER stress-related dysfunction and, despite restoring thapsigargin-induced translational repression, ISRIB did not mitigate thapsigargin-induced ATF4 expression.

### ISRIB pharmacokinetics and tolerability in C57BL/6J mice *in vivo*

**Figure 4.**
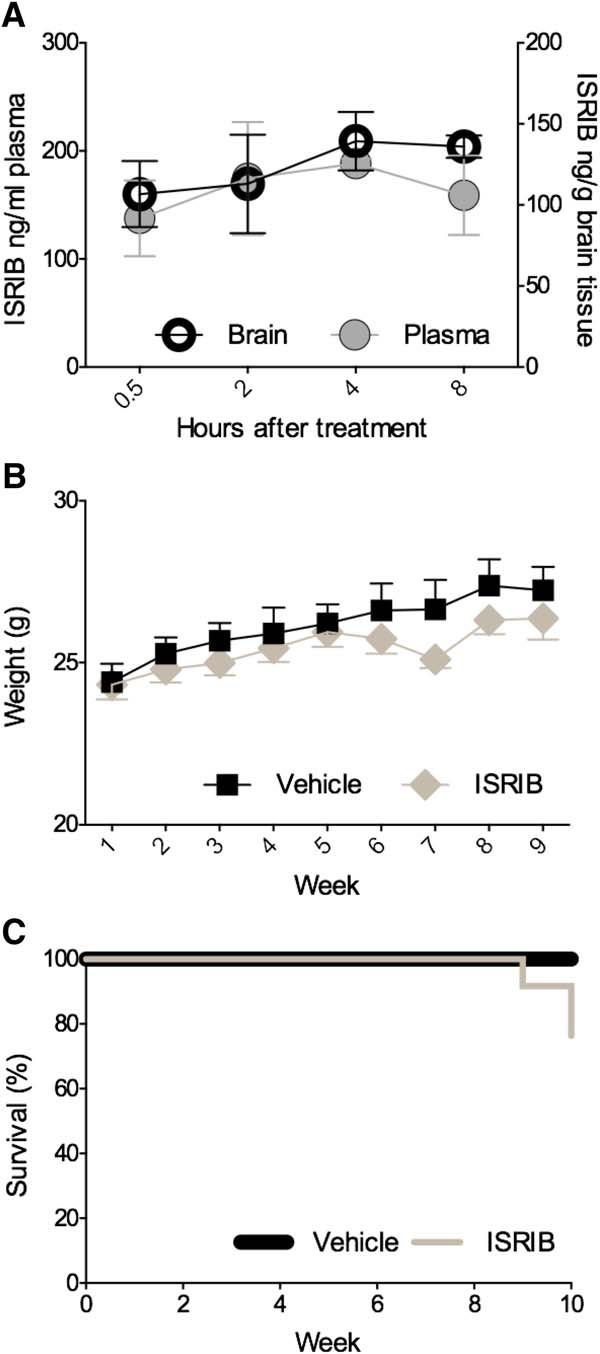
Peripherally administered ISRIB crosses the BBB and prolonged administration is well tolerated by C57BL/6J mice. ***A***, ISRIB concentration over time in brain and plasma of five-month-old C57BL/6J mice after a single intraperitoneal injection (5 mg/kg). Plasma collected at 0.5 h (*n* = 4), 2 h (*n* = 4), 4 h (*n* = 3), and 8 h (*n* = 4) after injection. Brain tissue collected at 0.5 h (*n* = 4), 2 h (*n* = 4), 4 h (*n* = 4), and 8 h (*n* = 4) after injection. ***B***, Body weights of mice receiving daily injections of vehicle or ISRIB for nine weeks. Vehicle, *n* = 6; ISRIB, *n* = 6. ***C***, Survival rates of vehicle or ISRIB-treated mice. Error bars indicate SEM.

Before examining the role of ER stress and its modulation in PS19 and APP^Swe^ mice, we needed to ensure that ISRIB crossed the blood-brain barrier (BBB) and that prolonged, once daily dosing could be tolerated. To determine if ISRIB permeated the brain, C57BL/6J mice received a single intraperitoneal injection of vehicle of ISRIB (5 mg/kg). Brain and plasma were collected at five time points following the injection: immediately (data not shown-ISRIB undetectable), 0.5, 2, 4, and 8 h. As shown in Figure 4*A*, ISRIB was successfully detected in brain and plasma at all collection points. ISRIB concentration could not be predicted by time, neither in brain (4.099*h + 108.9, *R*^2^ = 0.08998) nor plasma (1.564*h + 157.7, *R*^2^ = 0.004507). Secondary analysis by two-way ANOVA confirmed there was no effect of collection time (*F*_(3,23)_ = 0.6006, *p* = 0.6212) or collection source (*F*_(1,23)_ = 3.539, *p* = 0.0726) on ISRIB concentration. To ensure chronic ISRIB administration did not have any adverse effects on body weight or mortality *in vivo*, C57BL/6J mice received one intraperitoneal injection per day of vehicle or ISRIB (5 mg/kg) for nine weeks. The effect of prolonged, once daily ISRIB administration in C57BL/6J mice on body weight is shown in Figure 4*B*. The main effect of time on body weight (*F*_(8,80)_ = 20.26, *p* < 0.0001) was significant; however, the effect of treatment on body weight (*F*_(1,10)_ = 0.8533, *p* = 0.3774) was not (1 × 9 SPANOVA). The effect of ISRIB on mortality in C57BL/6J mice is shown in Figure 4*C*. No difference in the survival distribution was found between mice treated with vehicle and mice treated with ISRIB (Mantel-Cox; χ^2^ = 1.000, *p* = 0.3173). Together, these results confirmed that ISRIB permeated the brain and that chronic, once daily dosing was well tolerated by C57BL/6J mice.

### Tolerability to prolonged ISRIB administration in PS19 mice *in vivo*

**Figure 5.**
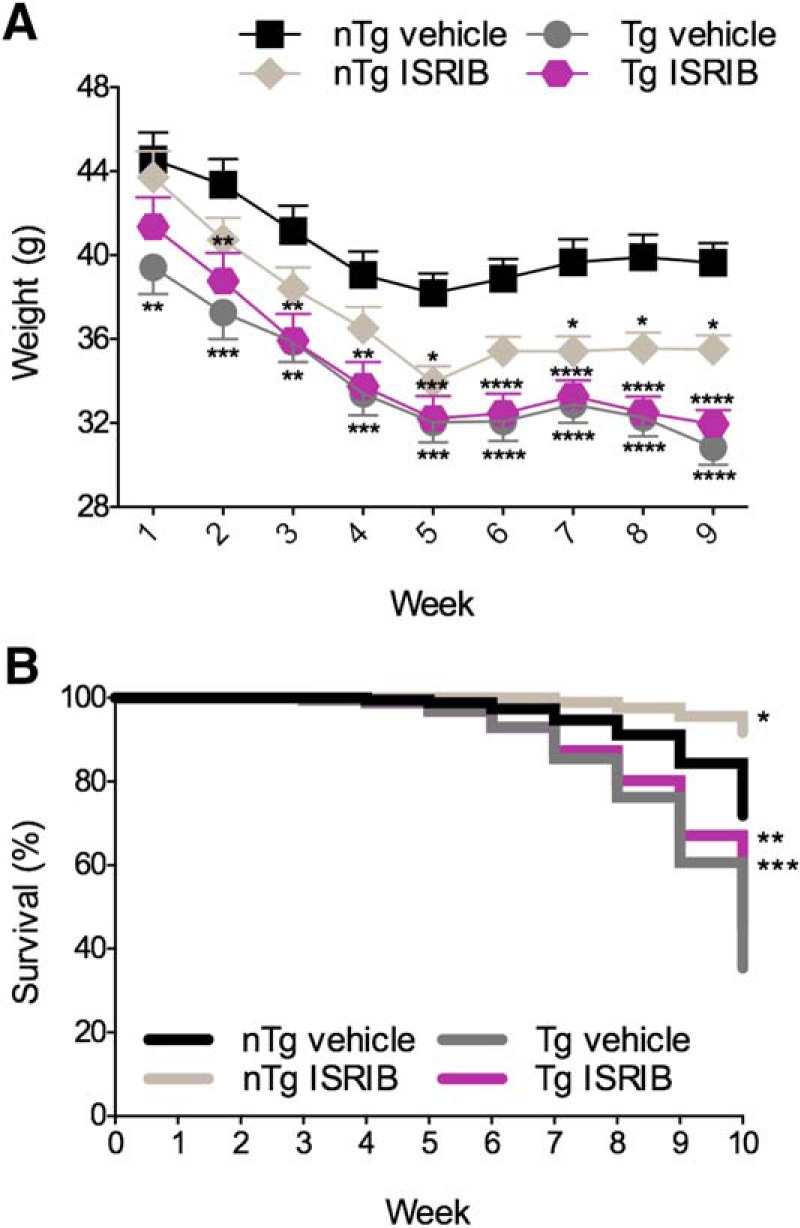
Effects of genotype and treatment on body weight and mortality in PS19 mice. ***A***, Tg mice have lower body weights compared to nTg mice. Daily administration of ISRIB reduced body weight in nTg mice over the course of nine weeks. ***B***, Tg mice have reduced survival rates compared to nTg mice. ISRIB improved survival in nTg mice. nTg vehicle, *n* = 27; nTg ISRIB, *n* = 25; Tg vehicle, *n* = 15; Tg ISRIB, *n* = 25. Asterisks indicate comparisons to nTg vehicle. Error bars indicate SEM; *p < 0.05, **p < 0.01, ***p < 0.001, ****p < 0.0001.

To investigate the role of ER stress in the AD-like manifestations observed in PS19 mice, nTg and Tg mice received once daily injections of either vehicle or ISRIB (5 mg/kg) for nine weeks while undergoing behavioral testing. The effects of prolonged, once daily administration of vehicle or ISRIB on body weight and mortality are shown in Figure 5. The effects of genotype and treatment on body weight are shown in Figure 5*A*. The main effects of time (*F*_(8,784)_ = 104.4, *p* < 0.0001) and group (i.e., genotype and treatment; *F*_(3,98)_ = 11.42, *p* < 0.0001) as well as their interaction (*F*_(24,784)_ = 1.722, *p* = 0.0173) were significant (6 × 9 SPANOVA). Compared to nTg mice treated with vehicle, nTg mice treated with ISRIB weighed significantly less on weeks 5, 7, 8, and 9 (Bonferroni’s MCT, *p* < 0.05). Compared to nTg mice treated with vehicle, Tg mice treated with vehicle weighed significantly less for the duration of the study (Bonferroni’s MCT; weeks 1 and 3, *p* < 0.01; week 2, 4, and 5, *p* < 0.001; weeks 6–9, *p* < 0.0001). Compared to nTg mice treated with vehicle, Tg mice treated with ISRIB weighed significantly less on all but week 1 (Bonferroni’s MCT; weeks 2–4, *p* < 0.01; week 5, *p* < 0.001; weeks 6–9, *p* < 0.0001). The effects of genotype and treatment on survival rate in PS19 mice are shown in Figure 5*B*. Compared to nTg mice treated with vehicle, nTg mice treated with ISRIB had a significantly higher survival rate (Mantel-Cox; χ^2^ = 6.535, *p* = 0.0106). Compared to nTg mice treated with vehicle, Tg mice treated with vehicle (Mantel-Cox; χ^2^ = 12.91, *p* = 0.0003) and Tg mice treated with ISRIB (Mantel-Cox; χ^2^ = 7.841, *p* = 0.0051) had significantly lower survival rates. There were no differences in the survival distribution of Tg mice treated vehicle and Tg mice treated with ISRIB (Mantel-Cox; χ^2^ = 0.6417, *p* = 0.4231). These results confirm that PS19 Tg mice exhibit phenotypically reduced body weight (López-González et al., 2015) and increased mortality (Yoshiyama et al., 2007) as previously reported. Based on these outcomes, prolonged, once daily administration of ISRIB was well tolerated by PS19 mice. These data provide assurance that behavioral outcomes, subsequently described, can be attributed to inherent genotype-dependent behavioral deficits and are not likely the result of a negative visceral reaction to the compounds used presently.

### Effects of genotype and eIF2B modulation on behavioral outcomes in PS19 mice *in vivo*

**Figure 6.**
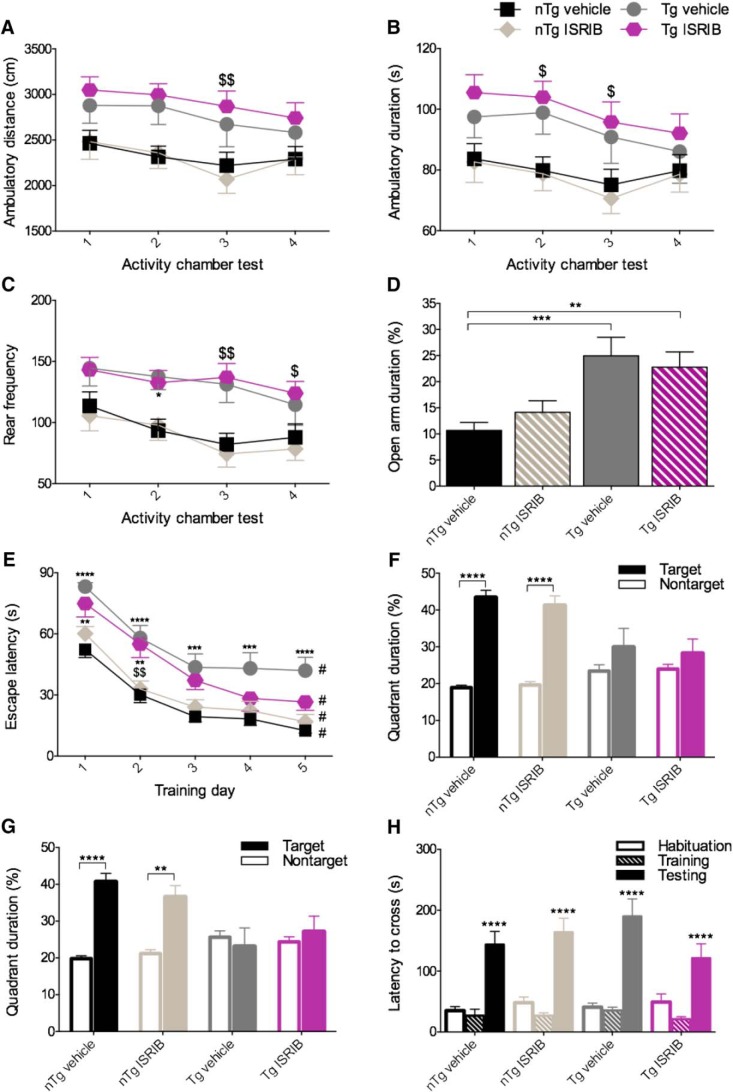
Despite severe behavioral impairments, ISRIB provides modest restoration of spatial acquisition deficits in PS19 Tg mice. ***A***–***C***, Tg mice exhibit a characteristically hyperactive phenotype. ***A***, Quantification of ambulatory distance. Tg mice treated with ISRIB ambulate a greater distance on AC test 3 compared to nTg mice treated with ISRIB. ***B***, Quantification of ambulatory duration. Tg mice treated with ISRIB spend more time moving on AC tests 2 and 3 compared to nTg mice treated with ISRIB. ***C***, Quantification of rear frequency. Tg mice treated with vehicle rear more frequently on AC test 2 compared to nTg mice treated with vehicle. Tg mice treated with ISRIB rear more frequently on AC tests 3 and 4 compared to nTg mice treated with ISRIB. nTg vehicle, *n* = 27; nTg ISRIB, *n* = 25; Tg vehicle, *n* = 25; Tg ISRIB, *n* = 25. ***D***, Tg mice exhibit diminished anxiety-like behavior. Tg mice treated with vehicle or ISRIB spend more time in the open arms of the EPM compared to nTg mice treated with vehicle. nTg vehicle, *n* = 26; nTg ISRIB, *n* = 25; Tg vehicle, *n* = 24; Tg ISRIB, *n* = 22. ***E***–***G***, Tg mice display impaired spatial learning and memory with modest restoration of acquisition by ISRIB. ***E***, Quantification of escape latency during the acquisition/training phase of the MWM. Tg mice treated with vehicle took longer to locate the platform on each day compared to nTg mice treated with vehicle. Tg mice treated with ISRIB took longer to locate the platform on days 1 and 2 compared to nTg mice treated with vehicle. Tg mice treated with ISRIB took longer to locate the platform on day 2 compared to nTg mice treated with ISRIB. All groups located the platform significantly faster on day 5 compared to day 1. ***F***, ***G***, Quantification of quadrant duration during the MWM probe test conducted 24 and 72 h after training. nTg mice treated with vehicle or ISRIB spent significantly more time in the target quadrant compared to the nontarget quadrant during the 24 h (***F***) and 72 h (***G***) probe tests. nTg vehicle, *n* = 23; nTg ISRIB, *n* = 24; Tg vehicle, *n* = 15; Tg ISRIB, *n* = 12. ***H***, Tg mice do not exhibit fear-based associative learning and memory deficits. Quantification of latency to cross during the habituation, training, and testing phases of the passive PA test are shown. All groups took significantly longer to cross during testing compared with the training phase. nTg vehicle, *n* = 24; nTg ISRIB, *n* = 23; Tg vehicle, *n* = 17; Tg ISRIB, *n* = 17. Asterisks indicate comparisons to nTg vehicle; $ indicate comparisons to nTg ISRIB; # indicate within group comparisons to trial 1. Error bars indicate SEM; $, *p < 0.05; $$, **p < 0.01, ***p < 0.001; #, ****p < 0.0001.

PS19 Tg mice have been reported to exhibit behavioral impairments including deficits in spatial and fear-based learning and memory (Takeuchi et al., 2011; Min et al., 2015; Lasagna-Reeves et al., 2016). To ensure that PS19 Tg mice expressed behavioral impairments consistent with those previously described and to investigate if ER stress was implicated in those deficits, PS19 mice underwent extensive behavioral testing (Fig. 6).

#### PS19 Tg mice exhibit locomotor hyperactivity

To assess locomotor activity, mice were tested in the activity chamber at four different time points throughout the study (Fig. 6*A–C*). The quantification of ambulatory distance is shown in Figure 6*A*. The main effects of activity chamber test number (two-way RM ANOVA; *F*_(3,294)_ = 6.455, *p* = 0.0003) and group (two-way RM ANOVA; *F*_(3,98)_ = 4.056, *p* = 0.0092) were significant. Compared to nTg mice treated with ISRIB, Tg mice treated with ISRIB ambulated a greater distance on test 3 (Bonferroni’s MCT, *p* < 0.01). The quantification of ambulatory duration is shown in Figure 6*B*. The main effects of activity chamber test number (two-way RM ANOVA; *F*_(3,294)_ = 5.847, *p* = 0.0007) and group (two-way RM ANOVA; *F*_(3,98)_ = 3.658, *p* = 0.0151) were significant. Compared to nTg mice treated with ISRIB, Tg mice treated with ISRIB had longer ambulatory durations on tests 2 and 3 (Bonferroni’s MCT, *p* < 0.05). The quantification of rearing frequency is shown in Figure 6*C*. The main effects of activity chamber test number (two-way RM ANOVA; *F*_(3,294)_ = 9.866, *p* < 0.0001) and group (two-way RM ANOVA; *F*_(3,98)_ = 5.879, *p* = 0.0010) were significant. Compared to nTg mice treated with vehicle, Tg mice treated with vehicle reared more frequently on test 2 (Bonferroni’s MCT, *p* < 0.05). Compared to nTg mice treated with ISRIB, Tg mice treated with ISRIB reared more frequently on tests 3 (Bonferroni’s MCT, *p* < 0.01) and 4 (Bonferroni’s MCT, *p* < 0.05). For the sake of analyzing the phenotype, PS19 Tg mice treated vehicle were compared to PS19 nTg mice treated with vehicle. The data from each AC test measure was pooled and analyzed using *t* tests (unpaired, two-tailed). Tg mice treated with vehicle ambulated a further distance (M = 2753, SD = 1107) than nTg mice treated with vehicle (M = 2336, SD = 711.5); *t*_(206)_ = 3.256, *p* = 0.0013. Tg mice treated with vehicle spent more time ambulating (M = 93.32, SD = 41.64) than nTg mice treated with vehicle (M = 79.60, SD = 25.69); *t*_(206)_ = 2.884, *p* = 0.0043. Finally, PS19 Tg mice treated with vehicle reared more frequently (M = 132.0, SD = 71.74) than nTg mice treated with vehicle (M = 94.31, SD = 54.25); *t*_(206)_ = 4.297, *p* < 0.0001. These results confirm that PS19 Tg mice express locomotor hyperactivity-consistent with their behavioral phenotype. A summary of all behavioral outcomes used for phenotype analysis in PS19 mice are provided in Table 3.

#### PS19 Tg mice exhibit diminished anxiety-like behavior

To examine anxiety-like behavior, PS19 mice were tested using the EPM (Fig. 6*D*). The main effect of genotype on time spent in the open arms (one-way ANOVA; *F*_(3,93)_ = 6.977, *p* = 0.0003) was significant. Compared to nTg mice treated with vehicle, Tg mice treated with vehicle (Bonferroni’s MCT, *p* < 0.001) and Tg mice treated with ISRIB (Bonferroni’s MCT, *p* < 0.01) spent a larger percentage of time in the open arms than the closed arms. These results confirm that PS19 Tg mice exhibit reduced anxiety-like behavior.

#### PS19 Tg mice exhibit spatial learning and memory deficits with modest restoration of spatial acquisition by ISRIB

To assess spatial learning and memory, mice were tested using the MWM (Fig. 6*E–G*). The main effects o*f* test day (*F*_(4,280)_ = 106.6, *p* < 0.0001) and group (*F*_(3,70)_ = 13.76, *p* < 0.0001) on escape latency (i.e., latency to locate and climb atop the platform) were significant (two-way RM ANOVA; Fig. 6*E*). Compared to nTg mice treated with vehicle, Tg mice treated with vehicle had longer escape latencies on all training days: days 1, 2, and 5 (Bonferroni’s MCT, *p* < 0.0001), days 3 and 4 (Bonferroni’s MCT, *p* < 0.001). Compared to nTg mice treated with vehicle, Tg mice treated with ISRIB had longer escape latencies on days 1 and 2 (Bonferroni’s MCT, *p* < 0.01). Compared to nTg mice treated with ISRIB, Tg mice treated with ISRIB took longer to escape on day 2 (Bonferroni’s MCT, *p* < 0.01). Performance in Tg mice treated with ISRIB was restored to near nTg levels on days 3, 4, and 5. To assess learning over time, performance on subsequent training days was compared within groups to performance on day 1. All groups performed significantly better on day 5 compared with day 1 (*p* < 0.0001), indicating that all groups acquired the task. To assess spatial memory recall, a probe test was administered 24 and 72 h following completion of the acquisition phase of the MWM. The percentage of time spent in the target and nontarget quadrants was assessed using paired-samples *t* tests. The effects of genotype and treatment on recall during the 24 h probe test are shown in Figure 6*F*. nTg mice treated with vehicle spent more time in the target quadrant (M = 43.3535, SD = 9.751) than the nontarget quadrant (M = 18.9039, SD = 3.252); *t*_(22)_ = 9.018, *p* = < 0.0001, and nTg mice treated with ISRIB spent more time in the target quadrant (M = 41.1725, SD = 13.06) than the nontarget quadrant (M = 19.6304, SD = 4.351); *t*_(23)_ = 6.063, *p* = < 0.0001. No significant differences in quadrant duration were found in Tg mice. The results of the 72 h probe test are shown in Figure 6*G*. nTg mice treated with vehicle spent more time in the target quadrant (M = 40.6565, SD = 11.04) than the nontarget quadrant (M = 19.813, SD = 3.691); *t*_(22)_ = 6.787, *p* < 0.0001, and nTg mice treated with ISRIB spent more time in the target quadrant (M = 36.5083, SD = 15.43) than the nontarget quadrant (M = 21.1833, SD = 5.140); *t*_(23)_ = 3.650, *p* = 0.0013. No significant differences in quadrant duration were found in Tg mice. To control for the confounding effect of vision impairments in PS19 mice, a visual platform test was administered 24 h later (data not shown) and the main effect of group on escape latency was found to be significant (one-way ANOVA; *F*_(3,70)_ = 15.87, *p* < 0.0001). As such, mice that met the exclusion criteria (as described in the methods) were retrogradely excluded from the study. These results indicate that ISRIB confers partial restoration of spatial learning deficits in Tg mice. By extension, these data suggest that ER stress, abbreviated presently by ISRIB, may be implicated in the behavioral manifestations observed in the PS19 model of tauopathy.

#### PS19 Tg mice do not exhibit impairments in fear-based learning and memory

To investigate fear-based learning and memory, the PA test was used (Fig. 6*H*). No significant differences were found between groups during the habituation (one-way ANOVA; *F*_(3,77)_ = 0.5574, *p* = 0.6448), training (one-way ANOVA; *F*_(3,78)_ = 0.5432, *p* = 0.6542), or recall (one-way ANOVA; *F*_(3,78)_ = 01.292, *p* = 0.2833) phases of the test. The main effect of task phase was found to be significant *F*_(2,234)_ = 78.50, *p* < 0.0001. Compared with the training phase, all groups took significantly longer to cross during the testing phase (*p* < 0.0001, Bonferroni’s MCT). These results indicate that all mice learned and recalled the test and that Tg mice do not display impaired fear-based learning and memory.

### Effects of genotype and eIF2B modulation on neuropathology in PS19 mice

**Figure 7.**
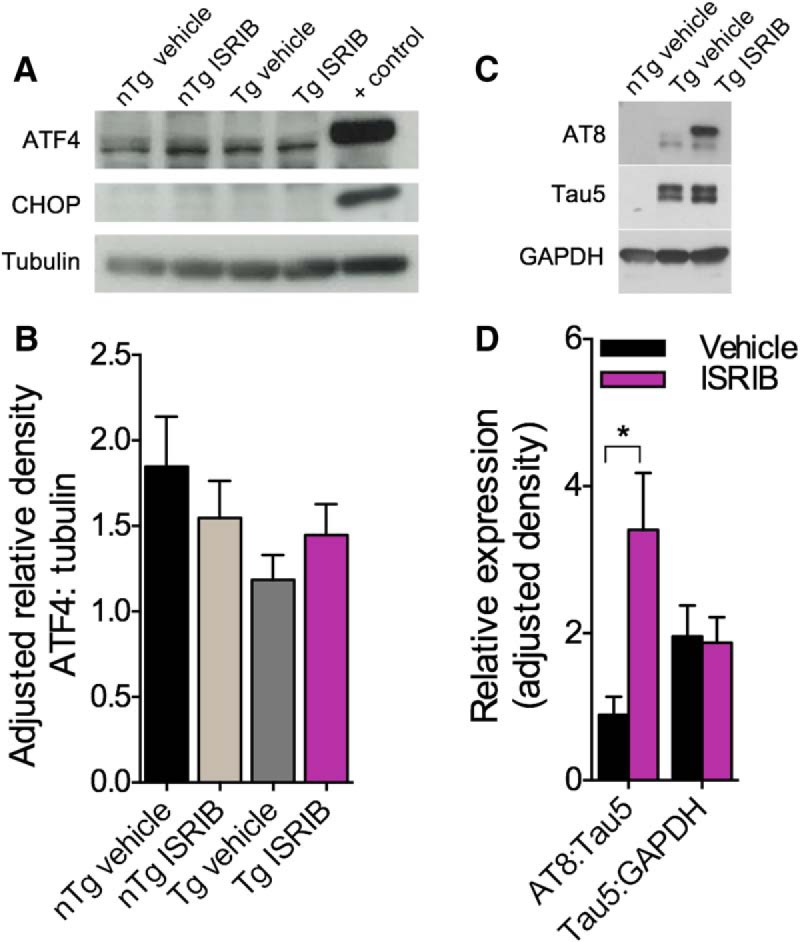
ER stress does not appear to be implicated in PS19 Tg neuropathology. ***A***, Representative immunoblot of cortical tissue homogenates from nTg and Tg mice probed using antibodies directed against ATF4 and CHOP. CHOP was detected only in PCN lysate derived from E17 Sprague Dawley rats treated with 1 μM thapsigargin, which served as a positive control (+ control). ***B***, Quantification of ATF4 normalized to tubulin. No significant differences in levels of ATF4 were found. nTg vehicle, *n* = 12; nTg ISRIB, *n* = 15; Tg vehicle, *n* = 6; Tg ISRIB, *n* = 8; + control (*n* = 2). ***C***, Representative immunoblot of hippocampal tissue homogenate from nTg and Tg mice probed using antibodies directed against p-tau (AT8) and total tau (Tau5). ***D***, Quantification of AT8 normalized to Tau5 and Tau5 normalized to GAPDH. Tg mice treated with ISRIB have significantly more AT8 compared to Tg mice treated with vehicle. Tg + vehicle, *n* = 6; Tg + ISRIB, *n* = 8. Error bars indicate SEM; *p < 0.05.

**Figure 8.**
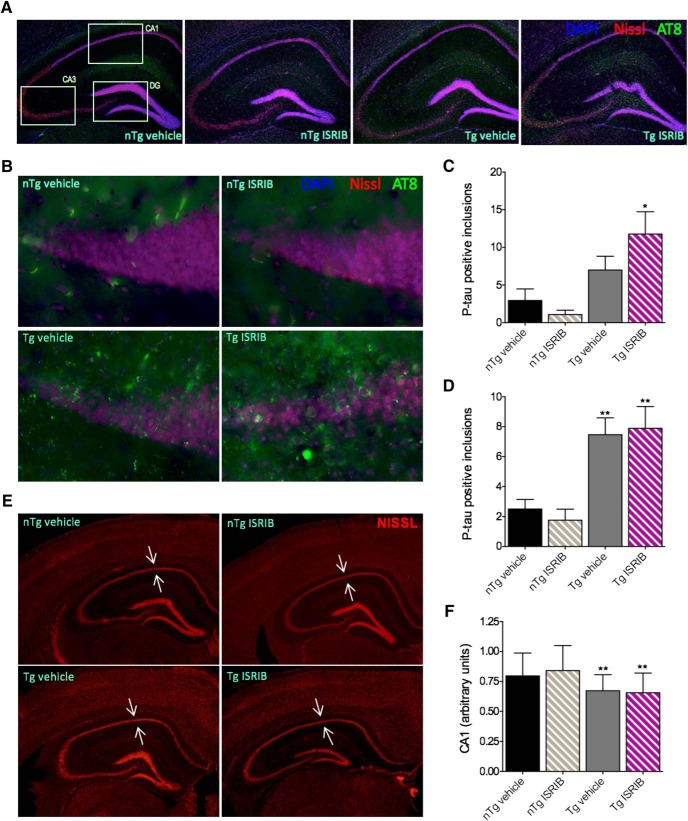
PS19 Tg mice have increased p-tau and evidence of hippocampal degeneration. ***A***, Representative photomicrographs (5×) of the hippocampus in nTg and Tg mice treated with vehicle or ISRIB. Sections stained using DAPI, fluoro-Nissl, and antibody directed at p-tau (AT8). Boxes indicate the ROIs. ***B***, Representative photomicrographs (40×) of the endal limb of the DG in nTg and Tg mice treated with vehicle or ISRIB. Sections stained using DAPI, fluoro-Nissl, and antibody directed at p-tau (AT8). ***C***, Quantification of AT8 in the DG. Tg mice treated with ISRIB have an increased number of p-tau-immunoreactive inclusions in the DG compared to nTg mice treated with vehicle. Error bars indicate SEM; *p < 0.05. ***D***, Quantification of AT8 in the hippocampus. Tg mice have an increased number of p-tau-immunoreactive inclusions throughout CA1, CA3, and the DG compared to nTg mice treated with vehicle. Error bars indicate SEM; **p < 0.01. ***E***, Representative photomicrographs (2.5×) of brain sections from nTg and Tg mice treated with vehicle or ISRIB. Arrows indicate the pyramidal cell layer of CA1. Sections stained using fluoro-Nissl. ***F***, Quantification of CA1 layer thickness. Tg mice have significant reductions in CA1 compared to nTg mice treated with vehicle. nTg vehicle, *n* = 7; nTg ISRIB, *n* = 7; Tg vehicle, *n* = 7; Tg ISRIB, *n* = 7. Error bars indicate SD; **p < 0.01.

PS19 Tg mice develop a robust neuropathology characterized by aberrant accumulations of hyper-phosphorylated tau and neuronal loss (Yoshiyama et al., 2007) and components of the PERK pathway may be implicated in this pathogenesis (Hoozemans et al., 2009; Salminen et al., 2009; Ho et al., 2012) and possibly contribute to behavioral impairments. Because a modest restoration of spatial memory acquisition was observed in Tg mice treated with ISRIB, we were interested to assess levels of ER stress-related markers ex vivo. On completion of behavioral testing, PS19 mice were sacrificed and their brains assessed for levels of ATF4, CHOP, and the development of AD-like neuropathology. The results of these analyses are shown in Figures 7, 8. Immunoblots of cortical tissue homogenates were probed for ATF4, CHOP, and tubulin (internal loading control; Fig. 7*A*). Lysate from E17 rat PCNs treated with thapsigargin was used as a positive control (+ control). CHOP was undetectable in PS19 mice (Fig. 7*A*) and no significant differences in levels of ATF4 were found (one-way ANOVA; *F*_(3,37)_ = 1.018, *p* = 0.3958; Fig. 7*B*). Immunoblots of hippocampal homogenates were probed for p-tau (AT8), total tau (Tau5), and GAPDH (internal loading control; Fig. 7*C*). Student’s *t* test revealed that Tg mice treated with ISRIB had more p-tau in the hippocampus (M = 0.8933, SD = 0.5938) than Tg mice treated with vehicle (M = 3.406, SD = 2.201); *t*_(12)_ = 2.699, *p* = 0.0193 (Fig. 7*D*). No difference was found in levels of Tau5 (M = 1.957, SD = 1.109) or GAPDH (M = 1.874, SD = 0.9811); *t*_(13)_ = 0.1546, *p* = 0.8795 (Fig. 7*D*). To determine if the development of tau pathology was region specific, IHC was performed and the regions of interest (ROIs) are indicated in Figure 8*A* along with 5× photomicrographs of the hippocampus in nTg and Tg mice, treated with and without ISRIB. Representative photomicrographs (40×) of the dentate gyrus (DG) are shown in Figure 8*B*. A main effect of group on the number of p-tau positive inclusions in the DG (one-way ANOVA; *F*_(3,24)_ = 6.165, *p* = 0.0029) was found to be significant (Fig. 8*C*). Compared to nTg mice treated with vehicle, Tg mice treated with ISRIB (Bonferroni’s MCT, *p* < 0.05) had significantly more p-tau in the DG. A significant main effect of group was also found on levels of p-tau in CA1 of the hippocampus (data not shown; one-way ANOVA; *F*_(3,24)_ = 3.213, *p* = 0.0408). While the results were not significant, Tg mice also had more p-tau in CA3 compared to nTg mice treated with vehicle (data not shown; one-way ANOVA; *F*_(3,24)_ = 2.723, *p* = 0.0667). The total number of p-tau positive inclusions summed across all regions of the hippocampus was found to be significant (Fig. 8*D*; one-way ANOVA; *F*_(3,80)_ = 9.547, *p* < 0.0001). Compared to nTg mice treated with vehicle, Tg mice treated with vehicle or ISRIB had increased levels of p-tau (Bonferroni’s MCT, *p* < 0.01) throughout the hippocampus. In addition, evidence of neuronal loss and hippocampal atrophy was observed in Tg mice. Representative photomicrographs (2.5×) of the hippocampus stained with fluoro-Nissl are shown in Figure 8*E*. Arrows indicate the pyramidal cell layer of CA1. The main effect of group on CA1 pyramidal cell layer thickness was found to be significant (one-way ANOVA; *F*_(3,164)_ = 11.28, *p* < 0.0001; Fig. 8*F*). Compared to nTg mice treated with vehicle, Tg mice treated with vehicle or ISRIB had significant thinning of the CA1 pyramidal cell layer (Bonferroni’s MCT, *p* < 0.01) to indicate neuronal loss. Together, these results confirm that PS19 Tg mice develop a significant neuropathology to include increased p-tau, and hippocampal atrophy indicative of neuronal loss. Interestingly, PS19 mice showed no evidence of ER stress-related increases in ATF4 or CHOP. Paradoxically, Tg mice treated with ISRIB, despite improved spatial memory acquisition, developed a worsened p-tau neuropathology. While no evidence of ER stress was found in this study, these data suggest that antagonism of eIF2B using ISRIB may restore translational repression in the PS19 mouse model of AD leading to an increase in tau phosphorylation.

### Tolerability of APP^Swe^ mice to ISRIB administration *in vivo*

**Figure 9.**
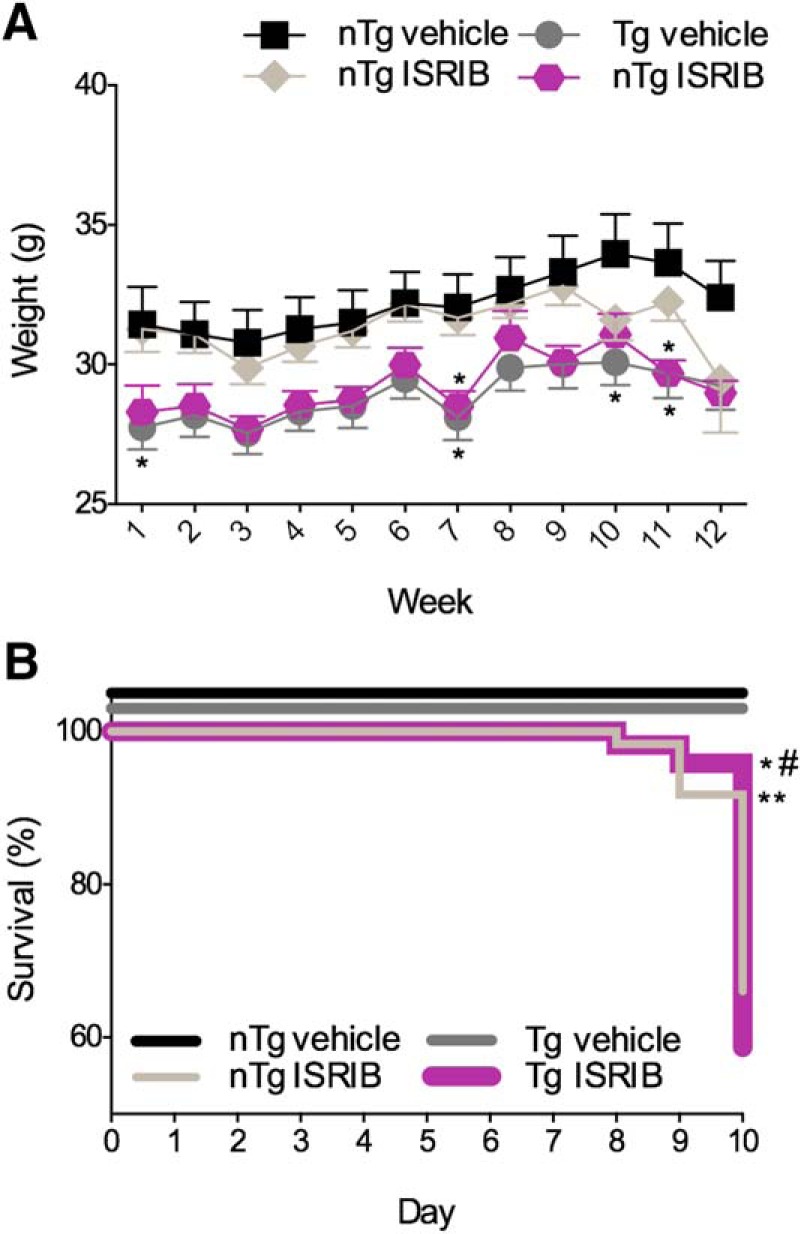
Effects of genotype and treatment on body weight and mortality in APP^Swe^ mice. ***A***, Tg mice have reductions in body weight compared with nTg mice. ***B***, ISRIB reduced survival in nTg and Tg mice by the eighth day of treatment. nTg vehicle, *n* = 14; nTg ISRIB, *n* = 15; Tg vehicle, *n* = 15; Tg ISRIB, *n* = 14. Asterisks indicate comparisons to nTg vehicle; # indicate comparisons to Tg vehicle. Error bars indicate SEM; *p < 0.05; #, **p < 0.01.

Next, we investigated the role of ER stress and eIF2B modulation on the AD-like manifestations observed in the APP^Swe^ mouse model of AD. The effects of genotype and treatment on body weight and survival in APP^Swe^ mice are shown in Figure 9. The same dosing paradigm used for C57BL/6J and PS19 mice, once daily administration of vehicle or ISRIB (5 mg/kg), resulted in significant mortality in APP^Swe^ mice by the eighth day of treatment. As such, the protocol was modified and mice received daily injections of vehicle or ISRIB (0.25 or 2.5 mg/kg) only on behavioral test days. The effects of treatment on body weight in nTg and Tg mice are shown in Figure 9*A*. The main effects of week (*F*_(11,594)_ = 18.84, *p* < 0.0001) and group (*F*_(3,54)_ = 3.896, *p* = 0.0136) as well as their interaction (*F*_(33,594)_ = 1.544, *p* = 0.0284) were significant (6 × 12 SPANOVA). Compared to nTg mice treated with vehicle, Tg mice treated with vehicle weighed less on weeks 1, 7, 10, and 11 (Bonferroni’s MCT, *p* < 0.05). Compared to nTg mice treated with vehicle, Tg mice treated with ISRIB weighed less on weeks 7 and 11 (Bonferroni’s MCT, *p* < 0.05). The effects of genotype and treatment on mortality rate are shown in Figure 9*B*. Compared to nTg mice treated with vehicle, nTg mice treated with ISRIB had a lower survival rate (Mantel-Cox; χ^2^ = 6.846, *p* = 0.0089). Compared to nTg mice treated with vehicle, Tg mice treated with vehicle (Mantel-Cox; χ^2^ = 6.480, *p* = 0.0109) had a lower survival rate. Finally, compared to Tg mice treated with vehicle, Tg mice treated with ISRIB had a lower survival rate (Mantel-Cox; χ^2^ = 6.923, *p* = 0.0085). Together, these data indicate that APP^Swe^ Tg mice have slightly lower body weights compared to nTg mice and that modulation of components in the PERK-eIF2α pathway may have adverse effects in this mouse model of amyloidosis.

### Effects of genotype and eIF2B modulation on behavioral outcomes in APP^Swe^ mice *in vivo*

**Figure 10.**
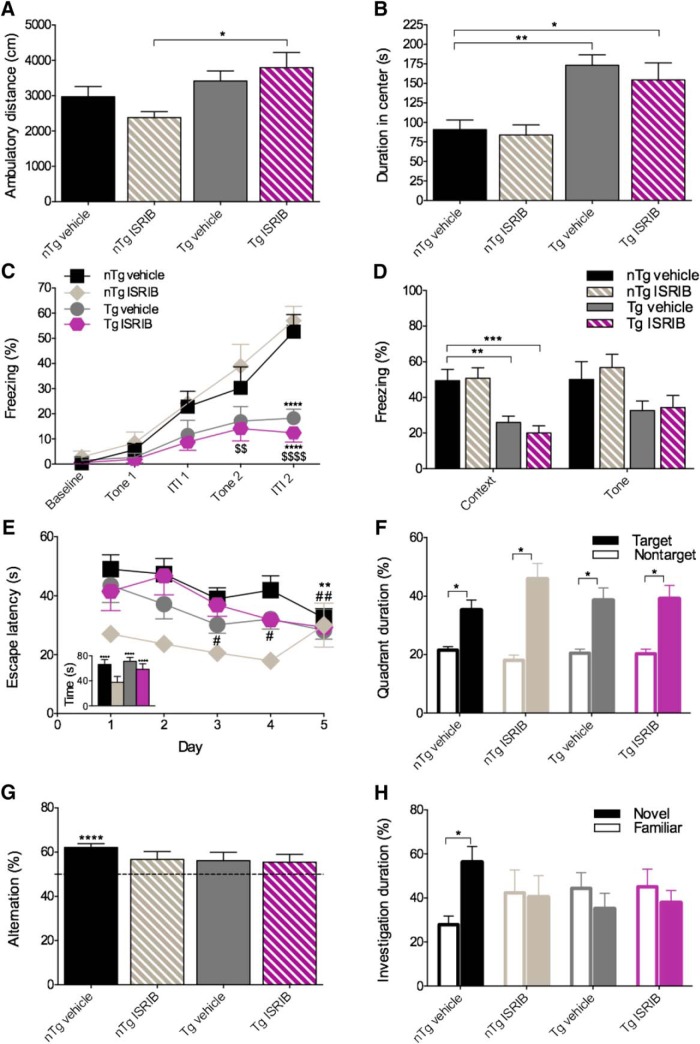
APP^Swe^ Tg mice exhibit learning and memory deficits and behavioral impairments. ***A***, ***B***, Tg mice display locomotor hyperactivity. ***A***, Quantification of ambulatory distance. Tg mice treated with ISRIB ambulate a greater distance compared to nTg mice treated with ISRIB. ***B***, Quantification of ambulatory duration in center. Tg mice spend more time in the center of the AC compared to nTg mice treated with vehicle. nTg vehicle, *n* = 14; nTg ISRIB, *n* = 13 (*n* = 15 for center duration); Tg vehicle, *n* = 14; Tg ISRIB, *n* = 14. ***C***, ***D***, Tg mice exhibit deficits in fear-based learning and memory. ***C***, Quantification of freezing behavior during the acquisition phase of the fear conditioning paradigm. Tg mice treated with ISRIB freeze less during tone 2 compared to nTg mice treated with ISRIB. During ITI2, Tg mice treated with vehicle or ISRIB freeze less compared to nTg mice treated with vehicle, and Tg mice treated with ISRIB freeze less compared to nTg mice treated with ISRIB. ***D***, Quantification of freezing behavior during context- or cue-based retrieval. Tg mice freeze significantly less compared to nTg mice treated with vehicle during context-based retrieval. No significant differences were found in time spent freezing to tone. nTg vehicle, *n* = 14; nTg ISRIB, *n* = 15; Tg vehicle, *n* = 15; Tg ISRIB, *n* = 14. Asterisks indicate comparisons to nTg vehicle, $ indicate comparisons to nTg ISRIB. ***E***, ***F***, Tg mice do not display deficits in spatial learning and memory. ***E***, Quantification of escape latency during the second MWM acquisition phase and reversal trial (inset). nTg mice treated with vehicle located the platform significantly faster on day 5 compared with day 1. Tg mice treated with vehicle located the platform significantly faster on days 3–5 compared with day 1. nTg mice treated with vehicle and Tg mice treated with vehicle or ISRIB took longer to locate the platform on the reversal trial (inset) compared with day 5. Additional between group comparisons were made for each training day and no significant differences were found; # indicates comparisons made within group to trial 1. ***F***, Quantification of escape latency during the second MWM probe trial. All groups spent significantly more time in the target quadrant compared with the nontarget quadrant. nTg vehicle, *n* = 10; nTg ISRIB, *n* = 4; Tg vehicle, *n* = 11; Tg ISRIB, *n* = 6. ***G***, ***H***, Tg mice exhibit phenotypic deficits in spatial working memory and short-term recognition memory. ***G***, Quantification of percentage alternation during the Y-maze. nTg mice treated with vehicle had a significantly greater percentage alternation compared with 50% chance alternation. No significant differences were found between groups in total number of entries or percentage of correct entries in the Y-maze (data not shown). nTg vehicle, *n* = 14; nTg ISRIB, *n* = 11; Tg vehicle, *n* = 15; Tg ISRIB, *n* = 10. ***H***, Quantification of percentage of time spent investigating the novel or familiar object during the NOR test. nTg mice treated with vehicle spent more time investigating the novel object compared with the familiar object. nTg vehicle, *n* = 12; nTg ISRIB, *n* = 10; Tg vehicle, *n* = 15; Tg ISRIB, *n* = 10. Error bars indicate SEM; #, *p < 0.05; ##, $$, **p < 0.01, ***p < 0.001; $$$$, ****p < 0.0001.

APP^Swe^ Tg mice have been found to express an AD-like age-dependent phenotype characterized by learning and memory impairments (Hsiao et al., 1996). To ensure that APP^Swe^ Tg mice expressed behavioral impairments consistent with those previously reported and to investigate if eIF2B modulation using ISRIB could restore these deficits, APP^Swe^ mice underwent extensive behavioral testing and the results are shown in Figure 10. A summary of all behavioral outcomes analyzed for phenotype in APP^Swe^ mice are provided in Table 4.

#### APP^Swe^ Tg mice exhibit locomotor hyperactivity

To determine the effects of genotype and treatment on locomotor activity, APP^Swe^ mice were tested in the activity chamber (Fig. 10*A*,*B*). A main effect of group on ambulatory distance (one-way ANOVA; *F*_(3,51)_ = 3.794, *p* = 0.0157) was found to be significant (Fig. 10*A*). Compared to nTg mice treated with ISRIB, Tg mice treated with ISRIB moved a greater distance (Bonferroni’s MCT, *p* = 0.0140). A main effect of group on center duration (one-way ANOVA; *F*_(3,53)_ = 8.377, *p* = 0.0001) was found to be significant (Fig. 10*B*). Compared to nTg mice treated with vehicle, Tg mice treated with vehicle (Bonferroni’s MCT, *p* = 0.0019) or ISRIB (Bonferroni’s MCT, *p* = 0.0239) spent more time in the center of the activity chamber.

#### APP^Swe^ Tg mice exhibit deficits in fear-based learning and memory

To assess the effects of genotype and treatment on fear-associated learning and memory, APP^Swe^ mice underwent fear conditioning followed by cued- or context-based retrieval testing (Fig. 10*C*,*D*). For fear conditioning, the main effects of trial (*F*_(4,216)_ = 50.36, *p* < 0.0001) and group (*F*_(3,54)_ = 8.473, *p* = 0.0001) as well as their interaction (*F*_(12,216)_ = 5.070, *p* < 0.0001) were significant (6 × 5 SPANOVA; Fig. 10*C*). Compared to nTg mice treated with vehicle, Tg mice treated with vehicle or ISRIB (Bonferroni’s MCT, *p* < 0.0001) froze less during ITI2. Compared to nTg mice treated with ISRIB, Tg mice treated with ISRIB froze less during tone 2 (Bonferroni’s MCT, *p* < 0.01) and ITI2 (Bonferroni’s MCT, *p* < 0.0001). The main effect of group on context-based retrieval (one-way ANOVA; *F*_(3,54)_ = 9.530, *p* < 0.0001) was significant (Fig. 10*D*). Compared to nTg mice treated with vehicle, Tg mice treated with vehicle (Bonferroni’s MCT, *p* = 0.0079) or ISRIB (Bonferroni’s MCT, *p* = 0.0008) froze less and while the results were not significant (*p* = 0.0662), Tg mice also froze less than nTg mice during cue-based retrieval.

#### APP^Swe^ Tg mice do not exhibit impairments in spatial learning and memory

To assess the effects of genotype and treatment on spatial learning and memory, APP^Swe^ mice were tested using the MWM (Fig. 10*E*,*F*). The main effect of test day (*F*_(4,480)_ = 5.602, *p* = 0.0002) on escape latency was found to be significant (4 × 4 SPANOVA; Fig. 10*E*). Compared with day 1, nTg mice treated with vehicle located the platform faster on day 5 (Bonferroni’s MCT, *p* = 0.0017). Compared with day 1, Tg mice treated with vehicle located the platform faster on days 3 (Bonferroni’s MCT, *p* = 0.0111), 4 (Bonferroni’s MCT, *p* = 0.0416), and 5 (Bonferroni’s MCT, *p* = 0.0026). No significant between-group differences were found on any of the test days (*p* = 0.0891). To ensure that mice had acquired the task, a reversal trial was conducted (Fig. 10*E*, inset) during which the location of the platform was moved. The effect of the reversal trial (*F*_(1,120)_ = 97.29, *p* < 0.0001) on escape latency was found to be significant (1 × 4 SPANOVA). Compared with day 5, all groups except nTg mice treated with ISRIB took longer to locate the platform on the reversal trial (Bonferroni’s MCT, *p* < 0.0001). During the probe test to assess spatial memory recall, percentage time spent in the target and nontarget quadrants was analyzed using paired-samples *t* tests (Fig. 10*F*). nTg mice treated with vehicle spent more time in the target quadrant (M = 35.25, SD = 10.97) than the nontarget quadrant (M = 21.60, SD = 3.658); *t*_(9)_ = 2.950, *p* = 0.0162 as did nTg mice treated with ISRIB (target: M = 45.86, SD = 10.76, nontarget: M = 18.062, SD = 3.586; *t*_(3)_ = 3.876, *p* = 0.0304), Tg mice treated with vehicle (target: M = 38.56, SD = 14.23, nontarget: M = 20.50, SD = 4.744; *t*_(10)_ = 3.157, *p* = 0.0102), and Tg mice treated with ISRIB (target: M = 39.07, SD = 11.36, nontarget: M = 20.33, SD = 3.786; *t*_(5)_ = 3.031, *p* = 0.0290).

#### APP^Swe^ Tg mice exhibit deficits in spatial working and recognition memory

To assess the effects of genotype and treatment on spatial working memory, the Y-maze was used (Fig. 10*G*). nTg mice treated with vehicle had a significantly greater percentage alternation compared with 50% chance alternation (M = 62.17, SD = 6.181); *t*_(12)_ = 7.099, *p* < 0.0001. No significant differences were found between groups in total number of entries or percentage of correct entries in the Y-maze (data not shown). The effects of genotype and treatment on recognition memory were assessed using the NOR test (Fig. 10*H*). Mice were also tested in the NOL test (data not shown); however, all groups failed to show learning. The percentage of time mice spent investigating the novel and non-novel objects was analyzed using paired-samples *t* tests. nTg mice treated with vehicle spent more time investigating the novel object (M = 56.24, SD = 24.80) compared with the familiar object (M = 27.85, SD = 13.56); *t*_(11)_ = 2.712, *p* = 0.0202. No significant differences were found between time spent with the novel versus familiar object in nTg mice treated with ISRIB (*p* = 0.9224), Tg mice treated with vehicle (*p* = 0.4998), or Tg mice treated with ISRIB (*p* = 0.5838).

Collectively, APP^Swe^ Tg mice exhibit locomotor hyperactivity, fear-based learning and memory deficits, impaired spatial working memory, and impaired recognition memory. Administration of ISRIB failed to restore any of these learning and memory deficits, indicating that ER stress-related dysfunction, and specifically the downstream effects of PERK-mediated eIF2 phosphorylation, may not mediate the manifestation of the behavioral deficits observed in the APP^Swe^ mouse model of AD.

## Discussion

Emerging evidence suggests that ER stress, and specifically changes in neuronal UPR, may be implicated in the pathogenesis of AD (Paschen and Mengesdorf, 2005a,b; Lindholm et al., 2006; Hoozemans et al., 2009; Scheper and Hoozemans, 2009). ATF4, an integral component of the UPR (Ron and Harding, 2012), is activated by ER stress and regulates the expression of the pro-apoptotic transcription factor CHOP (Ron and Habener, 1992; Averous et al., 2004). The present study aimed to investigate the involvement of ER stress in AD-like outcomes *in vitro*, as measured by ATF4 and CHOP, and *in vivo* using experimental AD models of tauopathy (MAPT P301S) and excessive amyloidosis (APP^Swe^). In addition, the present study investigated if pharmacological modulation of the eIF2B signaling cascade, via antagonism of the ISR, could ameliorate AD-like outcomes and implicate ER stress-related dysfunction in the disease pathogenesis of these models.

We hypothesized that ER stress would be involved in the AD-like pathology observed in these models and that ISRIB would mitigate the induction of ATF4 and CHOP *in vitro* and *in vivo*. We also hypothesized that ER stress-related dysfunction may contribute to the AD-like behavioral impairments observed in PS19 and APP^Swe^ Tg mice. Furthermore, we predicted that ISRIB would restore inherent deficits in learning and memory *in vivo* and curtail the neuropathology underlying these behavioral deficits.

In this study, we found that thapsigargin effectively induced ATF4, CHOP, and at higher concentrations, cytotoxicity, in PCNs derived from rats. Recently, ISRIB was found to mitigate the induction of ATF4 and CHOP in response to ER stress in HEK293 and U2OS cells (Sidrauski et al., 2013). We were able to partially corroborate those findings in rat PCNs. Despite a significant reduction in ATF4, ISRIB did not fully antagonize the effects of thapsigargin (i.e., CHOP) under the conditions used. Since ISRIB successfully mitigated the induction of ATF4 to indicate appropriate target engagement, we focused on ATF4 as a marker of ER stress.

As was observed in rat PCNs, thapsigargin also induced ATF4 in PCNs derived from APP^Swe^ nTg and Tg mice. Interestingly, basal levels of ATF4 were undetectable in both APP^Swe^ nTg and Tg cells, indicating that Tg cells show no evidence of constitutive ATF4 translation to indicate ER stress-related dysfunction. This was surprising since previously it was reported that eIF2α phosphorylation can occur in response to Aβ in cultured cells expressing Swe-APP (Kim et al., 2007). As such, it is possible that the Tg PCNs used in the present study had yet to express the Aβ pathology required to compromise PERK-eIF2α signaling and increase ER stress-related markers. We also found that thapsigargin induced comparable levels of ATF4 in nTg and Tg APP^Swe^ PCNs, indicating that cells isolated from Tg mice respond appropriately to ER stress. Presently, we were unable to mitigate the effects of thapsigargin on ATF4 induction using ISRIB in APP^Swe^ PCNs. While ISRIB seemed to be capable of reversing the translational inhibition induced by thapsigargin, this outcome appears to be independent of the ATF4 cascade. Under the same conditions, ISRIB reduced thapsigargin-induced ATF4 in rat PCNs cultured for 12–13 DIV. Because we observed a nonsignificant trend toward reduced ATF4 in mouse PCNs cultured for 11 DIV (data not shown), it is possible that a more pronounced reduction would be observed in cells cultured for longer periods of time or challenged with a reduced concentration of thapsigargin. Another possible explanation is that thapsigargin induces ATF4 through mechanisms other than deactivation of eIF2B. Despite our knowledge that ER stress can trigger the activation of ATF4, the functional role of ATF4 in the ER stress response is still not fully understood (Galehdar et al., 2010). Therefore, it is possible that downstream effects of the UPR and ISR can occur in the absence of ATF4 activity. In line with this possibility, a recent study found that deletion of ATF4 in liver was not required for the induction of the UPR transcription factor CHOP, but rather, ATF6 was found to be the primary inducer of CHOP (Fusakio et al., 2016).

Recently, the therapeutic effects of PERK modulation using ISRIB have been assessed using *in vivo* models of neurodegeneration (Halliday et al., 2015; Johnson and Kang, 2016). To investigate the role of ER stress and the effects of PERK pathway modulation in PS19 and APP^Swe^ *in vivo*, mice underwent extensive behavioral testing. In line with previous reports, PS19 Tg mice had reduced body weight, increased mortality and expressed a behavioral phenotype characterized by locomotor hyperactivity, diminished anxiety-like behavior (López-González et al., 2015), and behavioral impairments (Takeuchi et al., 2011; Min et al., 2015). Aside from a modest improvement in spatial memory acquisition in the MWM, pharmacological antagonism of the ISR using ISRIB was minimally effective at restoring the behavioral deficits observed in PS19 Tg mice. However, postmortem analysis revealed no evidence of ER stress-related induction in PS19 Tg mice, who had comparable levels of ATF4 in the hippocampus to nTg mice. This may again indicate a lack of target presence or activation, and therefore engagement, upstream of ATF4. For example, in prion-diseased mice with elevated ISR activity, ISRIB was found to exert therapeutic effects (Halliday et al., 2015). In this model, ISRIB prevented neuronal loss in the hippocampus, reduced levels of ATF4, and prevented the development of prion spongiform pathology. ISRIB also slowed the progression of prion disease by preventing the development of confirmatory signs including deficits in balance and motor coordination and sensorimotor impairment (Halliday et al., 2015). Another possibility is that the mechanisms that govern ER stress-related pathways and the development of AD-like neuropathologies are not conserved across animal models of AD or their *in vitro* counterparts. For example, while elevated levels of ATF4 have been reported in the hippocampus of APP-PS1 (Ma et al., 2013), 5xFAD (Devi and Ohno, 2013), and APOE4 mice (Segev et al., 2015), ATF4 was not detected in neither the cortex nor hippocampus of hAPP-J20 mice (Johnson and Kang, 2016).

Despite lack of evidence for ER stress-related dysfunction, PS19 Tg mice developed significant hippocampal atrophy evidenced by reductions in the pyramidal cell layer of CA1. Similarly, in human AD, ∼70% of neurons in CA1 of the hippocampus have been found to die during the progression of the disease (West et al., 1994). Previous studies employing the PS19 model have reported signs of synaptic dysfunction in CA1/CA3 (Yoshiyama et al., 2007), neuronal apoptosis in CA1, CA3, and the DG (López-González et al., 2015), and hippocampal atrophy (Min et al., 2015). In line with these previous reports, PS19 Tg mice also developed significant tau pathology. Paradoxically, and despite a slight improvement during the acquisition phase of the MWM, PS19 Tg mice treated with ISRIB had increased p-tau in the DG compared with Tg mice treated with vehicle. These data may suggest that reversal of eIF2α-mediated translational repression by ISRIB may lead to restoration of protein synthesis and as a consequence, an excessive hyper-phosphorylation of tau. Because ISRIB was previously found to improve behavioral function in healthy, WT mice, it is also possible that ISRIB exerts nootropic effects on other pathways to compensate for increased disease pathology. Another possibility is that modulation of the PERK pathway can affect behavioral function through targets other than those implicated in disease pathogenesis. For example, a recent study found that activation of mGluRs, through phosphorylation of eIF2α, induced long-term depression (LTD) by downregulating surface AMPAR density at synapses (Di Prisco et al., 2014) in the hippocampus. Furthermore, hippocampal LTD was found to be crucial for spatial learning of object-place recognition and spatial recognition of objects triggered LTD at Schaffer collateral–CA1 synapses in freely moving animals (Di Prisco et al., 2014). Similarly, increased LTD has been reported in the presence of Aβ oligomers in rat hippocampal slices (Shankar et al., 2008) and several animal models of AD have reported synaptic loss in regions proximal to Aβ plaques (Pozueta et al., 2013). Collectively, the loss of functional synapses may underlie the development of learning and memory deficits and behavioral impairments observed in human and animal models of AD (Lüscher and Huber, 2010). As such, the identification of additional markers of target engagement in Tg models of AD, particularly behavioral-related markers, would aid our understanding of disease pathogenesis and its effect on p-eIF2α-dependent LTD.

PS19 mice showed no adverse effects of prolonged, once daily ISRIB administration, yet a significant increase in mortality was observed in APP^Swe^ mice. This may be attributable to some yet unidentified off-target pharmacology. Other studies have reported significant reductions in body weight following long-term ISRIB administration (Halliday et al., 2015), indicating that ISRIB may have some level of toxicity. *In vivo*, APP^Swe^ Tg mice displayed behavioral impairments, including locomotor hyperactivity and learning and memory deficits (Hsiao et al., 1996; Jacobsen et al., 2006). We observed no therapeutic effect of ISRIB on these behavioral outcomes. However, previous research found no evidence of UPR activation or the induction of cell death pathways in APP^Swe^ mice (Lee et al., 2010b). In addition, a recent study found that ISRIB did not improve deficits in spatial learning and memory In hAPP-J20 Tg mice, nor did it enhance behavioral function in nTg mice (Johnson and Kang, 2016). Based on this research, and our lack of behavioral or *in vitro* evidence to support ER stress-related dysfunction in the disease pathogenesis of APP^Swe^ mice, we did not investigate further outcomes postmortem.

While ISRIB was minimally effective at improving the learning and memory performance and neuropathological outcomes in these mouse strains, several other compounds targeting the PERK-eIF2α pathway have been found to ameliorate dementia-like or ER stress-related outcomes (Boyce et al., 2005; Kim et al., 2008; Lee et al., 2010a; Radford et al., 2015). In Tg mice overexpressing the PS01L mutation, dysregulation of the PERK signaling pathway was associated with the onset of neurodegeneration (Radford et al., 2015). Treatment using a PERK inhibitor (GSK2606414) was reported to prevent further neuronal loss and reduced levels of phosphorylated tau (Radford et al., 2015). However, these mice also had elevated levels of ATF4, and phosphorylated PERK and eIF2α (Radford et al., 2015), providing a platform conducive for target engagement. However, in prion-diseased mice, treatment with GSK2606414 resulted in significant pancreatic toxicity (Moreno et al., 2013). Salubrinal, a drug that blocks the dephosphorylation of p-eIF2α, has been reported to enhance cell survival *in vitro* following ER stress (Boyce et al., 2005; Kim et al., 2008). Another study found that treatment with Aβ triggered the UPR in SK-N-SH human neuroblastoma cells, and selective activation of the PERK pathway using Salubrinal prevented Aβ-induced toxicity (Lee et al., 2010a). Collectively, these data suggest that the therapeutic outcomes of PERK-eIF2α pathway modulation are highly dependent on the component targeted in the pathway as well as the disease model in which it is assessed. Because ISRIB targets components downstream of p-eIF2α, this allows for the possibility that further upstream targets may be implicated in the disease pathogenesis of the models used presently.

Taken together, we have shown that modulation of eIF2B mitigated the induction of ATF4 in response to thapsigargin in PCNs derived from rats but not APP^Swe^ mice. While ISRIB exerted minimal therapeutic effects on the neuropathological and behavioral hallmarks observed in PS19 and APP^Swe^ mice, it is important to note that idiosyncrasies in ER stress-related markers were not observed. While it is possible that the therapeutic targets of ISRIB are not implicated or conserved in the AD-like pathology in the animal models examined in this study, these findings warrant further investigation. Future studies might examine the extent to which animal models recapitulate the mechanisms that give rise to AD-like neuropathology and its' behavioral correlates. In conclusion, understanding the functional role of ER stress in the pathogenesis of AD is a promising avenue toward the development of more targeted pharmaceuticals. The use of pharmacological tools, such as ISR antagonists, may expedite our understanding of this insidious disease.

## Synthesis

Reviewing Editor: Lorna Role, Stony Brook Decisions are customarily a result of the Reviewing Editor and the peer reviewers coming together and discussing their recommendations until a consensus is reached. When revisions are invited, a fact-based synthesis statement explaining their decision and outlining what is needed to prepare a revision will be listed below. The following reviewer(s) agreed to reveal their identity: Gordon Meares.

The referees are content with the revisions made -- both felt it was ready for publication “as is”

## References

[B1] Alzheimer’s Association (2013) Alzheimer’s disease facts and figures. Alzheimers Dement 9:208–245. 10.1016/j.jalz.2013.02.003 23507120

[B2] Ameri K, Harris AL (2008) Activating transcription factor 4. Int J Biochem Cell Biol 40:14–21. 10.1016/j.biocel.2007.01.020 17466566

[B3] Averous J, Bruhat A, Jousse C, Carraro V, Thiel G, Fafournoux P (2004) Induction of CHOP expression by amino acid limitation requires both ATF4 expression and ATF2 phosphorylation. J Biol Chem 279:5288–5297. 10.1074/jbc.M311862200 14630918

[B4] Boyce M, Bryant KF, Jousse C, Long K, Harding HP, Scheuner D, Kaufman RJ, Ma D, Coen DM, Ron D, Yuan J (2005) A selective inhibitor of eIF2alpha dephosphorylation protects cells from ER stress. Science 307:935–939. 10.1126/science.1101902 15705855

[B5] De Pietri Tonelli D, Mihailovich M, Di Cesare A, Codazzi F, Grohovaz F, Zacchetti D (2004) Translational regulation of BACE-1 expression in neuronal and non-neuronal cells. Nucleic Acids Res 32:1808–1817. 10.1093/nar/gkh348 15034149 PMC390341

[B6] Devi L, Ohno M (2010) Phospho-eIF2α level is important for determining abilities of BACE1 reduction to rescue cholinergic neurodegeneration and memory defects in 5XFAD mice. PLoS One 5 10.1371/journal.pone.0012974 PMC2944882 20886088

[B7] Devi L, Ohno M (2013) Deletion of the eIF2α kinase GCN2 fails to rescue the memory decline associated with Alzheimer’s disease. PLoS One 8:e77335. 10.1371/journal.pone.0077335 24146979 PMC3795630

[B8] Di Prisco GV, Huang W, Buffington SA, Hsu CC, Bonnen PE, Placzek AN, Sidrauski C, Krnjevic K, Kaufman RJ, Walter P, Costa-Mattioli M (2014) Translational control of mGluR-dependent long-term depression and object-place learning by eIF2α. Nat Neurosci 17:1073–1082. 10.1038/nn.3754 24974795 PMC4340591

[B9] Fusakio ME, Willy JA, Wang Y, Mirek ET, Al Baghdadi RJ, Adams CM, Anthony TG, Wek RC (2016) Transcription factor ATF4 directs basal and stress-induced gene expression in the unfolded protein response and cholesterol metabolism in the liver. Mol Biol Cell 27:1536–1551. 10.1091/mbc.E16-01-0039 26960794 PMC4850040

[B10] Galehdar Z, Swan P, Fuerth B, Callaghan SM, Park DS, Cregan SP (2010) Neuronal apoptosis induced by endoplasmic reticulum stress is regulated by ATF4-CHOP-mediated induction of the Bcl-2 homology 3-only member PUMA. J Neurosci 30:16938–16948. 10.1523/JNEUROSCI.1598-10.2010 21159964 PMC6634926

[B11] Gauthier S, Feldman HH, Schneider LS, Wilcock GK, Frisoni GB, Hardlund JH, Moebius HJ, Bentham P, Kook KA, Wischik DJ, Schelter BO, Davis CS, Staff RT, Bracoud L, Shamsi K, Storey JM, Harrington CR, Wischik CM (2016) Efficacy and safety of tau-aggregation inhibitor therapy in patients with mild or moderate Alzheimer's disease: a randomised, controlled, double-blind, parallel-arm, phase 3 trial. Lancet 388:2873–2884. 10.1016/S0140-6736(16)31275-2 PMC5164296 27863809

[B12] Halliday M, Radford H, Sekine Y, Moreno J, Verity N, le Quesne J, Ortori CA, Barrett DA, Fromont C, Fischer PM, Harding HP, Ron D, Mallucci GR (2015) Partial restoration of protein synthesis rates by the small molecule ISRIB prevents neurodegeneration without pancreatic toxicity. Cell Death Dis 6 10.1038/cddis.2015.49 PMC4385927 25741597

[B13] Harding HP, Zhang Y, Ron D (1999) Protein translation and folding are coupled by an endoplasmic-reticulum-resident kinase. Nature 397:271–274. 10.1038/16729 9930704

[B14] Harding HP, Zhang Y, Bertolotti A, Zeng H, Ron D (2000) Perk is essential for translational regulation and cell survival during the unfolded protein response. Mol Cell 5:897–904. 10882126 10.1016/s1097-2765(00)80330-5

[B15] Ho YS, Yang XF, Lau JCF, Hung CHL, Wuwongse S, Zhang QS, Wang JZ, Baum L, So KF, Chang RCC (2012) Endoplasmic reticulum stress induces tau pathology and forms a vicious cycle: implication in Alzheimer's disease pathogenesis. J Alzheimers Dis 28:839–854. 22101233 10.3233/JAD-2011-111037

[B16] Hoozemans JJ, Veerhuis R, Van Haastert ES, Rozemuller JM, Baas F, Eikelenboom P, Scheper W (2005) The unfolded protein response is activated in Alzheimer's disease. Acta Neuropathol 110:165–172. 10.1007/s00401-005-1038-0 15973543

[B17] Hoozemans JJM, van Haastert ES, Nijholt DAT, Rozemuller AJM, Eikelenboom P, Scheper W (2009) The unfolded protein response is activated in pretangle neurons in Alzheimer's disease hippocampus. Am J Pathol 174:1241–1251. 10.2353/ajpath.2009.080814 19264902 PMC2671357

[B18] Hsiao K, Chapman P, Nilsen S, Eckman C, Harigaya Y, Younkin S, Yang F, Cole G (1996) Correlative memory deficits, Abeta elevation, and amyloid plaques in transgenic mice. Science 274:99–102. 8810256 10.1126/science.274.5284.99

[B19] Iqbal K, Flory M, Khatoon S, Soininen H, Pirttila T, Lehtovirta M, Alafuzoff I, Blennow K, Andreasen N, Vanmechelen E, Grundke-Iqbal I (2005) Subgroups of Alzheimer's disease based on cerebrospinal fluid molecular markers. Ann Neurol 58:748–757. 10.1002/ana.20639 16247771

[B20] Jacobsen JS, Wu CC, Redwine JM, Comery TA, Arias R, Bowlby M, Martone R, Morrison JH, Pangalos MN, Reinhart PH, Bloom FE (2006) Early-onset behavioral and synaptic deficits in a mouse model of Alzheimer's disease. Proc Natl Acad Sci USA 103:5161–5166. 10.1073/pnas.0600948103 16549764 PMC1405622

[B21] Johnson EC, Kang J (2016) A small molecule targeting protein translation does not rescue spatial learning and memory deficits in the hAPP-J20 mouse model of Alzheimer's disease. PeerJ 4:e2565. 10.7717/peerj.2565 27781164 PMC5075699

[B22] Kim HS, Choi Y, Shin KY, Joo Y, Lee YK, Jung SY, Suh YH, Kim JH (2007) Swedish amyloid precursor protein mutation increases phosphorylation of eIF2alpha in vitro and in vivo. J Neurosci Res 85:1528–1537. 10.1002/jnr.21267 17393484

[B23] Kim I, Xu W, Reed JC (2008) Cell death and endoplasmic reticulum stress: disease relevance and therapeutic opportunities. Nat Rev Drug Discov 7:1013–1030. 10.1038/nrd2755 19043451

[B24] Lammich S, Schobel S, Zimmer AK, Lichtenthaler SF, Haass C (2004) Expression of the Alzheimer protease BACE1 is suppressed via its 5'-untranslated region. EMBO Rep 5:620–625. 10.1038/sj.embor.7400166 15167888 PMC1299076

[B25] Lasagna-Reeves CA, de Haro M, Hao S, Park J, Rousseaux MW, Al-Ramahi I, Jafar-Nejad P, Vilanova-Velez L, See L, De Maio A, Nitschke L, Wu Z, Troncoso JC, Westbrook TF, Tang J, Botas J, Zoghbi HY (2016) Reduction of Nuak1 decreases tau and reverses phenotypes in a tauopathy mouse model. Neuron 92:407–418. 10.1016/j.neuron.2016.09.022 27720485 PMC5745060

[B26] Lee DY, Lee KS, Lee HJ, Kim DH, Noh YH, Yu K, Jung HY, Lee SH, Lee JY, Youn YC, Jeong Y, Kim DK, Lee WB, Kim SS (2010a) Activation of PERK signaling attenuates Abeta-mediated ER stress. PLoS One 5:e10489. 10.1371/journal.pone.0010489 20463975 PMC2864758

[B27] Lee JH, Won SM, Suh J, Son SJ, Moon GJ, Park UJ, Gwag BJ (2010b) Induction of the unfolded protein response and cell death pathway in Alzheimer's disease, but not in aged Tg2576 mice. Exp Mol Med 42:386–394. 10.3858/emm.2010.42.5.040 20368688 PMC2877248

[B28] Lenna S, Trojanowska M (2012) The role of endoplasmic reticulum stress and the unfolded protein response in fibrosis. Curr Opin Rheumatol 24:663–668. 10.1097/BOR.0b013e3283588dbb 22918530 PMC3828639

[B29] Li J, Ni M, Lee B, Barron E, Hinton DR, Lee AS (2008) The unfolded protein response regulator GRP78/BiP is required for endoplasmic reticulum integrity and stress-induced autophagy in mammalian cells. Cell Death Differ 15:1460–1471. 10.1038/cdd.2008.81 18551133 PMC2758056

[B30] Lindholm D, Wootz H, Korhonen L (2006) ER stress and neurodegenerative diseases. Cell Death Differ 13:385–392. 10.1038/sj.cdd.4401778 16397584

[B31] López-González I, Aso E, Carmona M, Armand-Ugon M, Blanco R, Naudí A, Cabré R, Portero-Otin M, Pamplona R, Ferrer I (2015) Neuroinflammatory gene regulation, mitochondrial function, oxidative stress, and brain lipid modifications with disease progression in tau P301S transgenic mice as a model of frontotemporal lobar degeneration-tau. J Neuropathol Exp Neurol 74:975–999. 10.1097/NEN.0000000000000241 26360374

[B32] Lüscher C, Huber KM (2010) Group 1 mGluR-dependent synaptic long-term depression: mechanisms and implications for circuitry and disease. Neuron 65:445–459. 10.1016/j.neuron.2010.01.016 20188650 PMC2841961

[B33] Ma T, Trinh MA, Wexler AJ, Bourbon C, Gatti E, Pierre P, Cavener DR, Klann E (2013) Suppression of eIF2α kinases alleviates Alzheimer's disease-related plasticity and memory deficits. Nat Neurosci 16:1299–1305. 10.1038/nn.3486 23933749 PMC3756900

[B34] Marciniak SJ, Yun CY, Oyadomari S, Novoa I, Zhang Y, Jungreis R, Nagata K, Harding HP, Ron D (2004) CHOP induces death by promoting protein synthesis and oxidation in the stressed endoplasmic reticulum. Genes Dev 18:3066–3077. 10.1101/gad.1250704 15601821 PMC535917

[B35] Marciniak SJ, Garcia-Bonilla L, Hu J, Harding HP, Ron D (2006) Activation-dependent substrate recruitment by the eukaryotic translation initiation factor 2 kinase PERK. J Cell Biol 172:201–209. 10.1083/jcb.200508099 16418533 PMC2063550

[B36] Mihailovich M, Thermann R, Grohovaz F, Hentze MW, Zacchetti D (2007) Complex translational regulation of BACE1 involves upstream AUGs and stimulatory elements within the 5 ' untranslated region. Nucleic Acids Res 35:2975–2985. 10.1093/nar/gkm191 17439957 PMC1888809

[B37] Min SW, Chen X, Tracy TE, Li Y, Zhou Y, Wang C, Shirakawa K, Minami SS, Defensor E, Mok SA, Sohn PD, Schilling B, Cong X, Ellerby L, Gibson BW, Johnson J, Krogan N, Shamloo M, Gestwicki J, Masliah E, et al. (2015) Critical role of acetylation in tau-mediated neurodegeneration and cognitive deficits. Nat Med 21:1154–1162. 10.1038/nm.3951 26390242 PMC4598295

[B38] Moreno JA, Halliday M, Molloy C, Radford H, Verity N, Axten JM, Ortori CA, Willis AE, Fischer PM, Barrett DA, Mallucci GR (2013) Oral treatment targeting the unfolded protein response prevents neurodegeneration and clinical disease in prion-infected mice. Sci Transl Med 5:206ra138. 10.1126/scitranslmed.3006767 24107777

[B39] Mullane K, Williams M (2013) Alzheimer's therapeutics: continued clinical failures question the validity of the amyloid hypothesis-but what lies beyond? Biochem Pharmacol 85:289–305. 10.1016/j.bcp.2012.11.014 23178653

[B40] O'Connor T, Sadleir KR, Maus E, Velliquette RA, Zhao J, Cole SL, Eimer WA, Hitt B, Bembinster LA, Lammich S, Lichtenthaler SF, Hebert SS, De Strooper B, Haass C, Bennett DA, Vassar R (2008) Phosphorylation of the translation initiation factor eIF2alpha increases BACE1 levels and promotes amyloidogenesis. Neuron 60:988–1009. 19109907 10.1016/j.neuron.2008.10.047 PMC2667382

[B41] Paschen W, Mengesdorf T (2005a) Endoplasmic reticulum stress response and neurodegeneration. Cell Calcium 38:409–415. 10.1016/j.ceca.2005.06.019 16087231

[B42] Paschen W, Mengesdorf T (2005b) Cellular abnormalities linked to endoplasmic reticulum dysfunction in cerebrovascular disease–therapeutic potential. Pharmacol Ther 108:362–375. 16140387 10.1016/j.pharmthera.2005.05.008

[B43] Pozueta J, Lefort R, Shelanski ML (2013) Synaptic changes in Alzheimer's disease and its models. Neuroscience 251:51–65. 10.1016/j.neuroscience.2012.05.050 22687952

[B44] Prince MWA, Guerchet M, Gemma C, Wu TY, Prina M (2015) World Alzheimer report 2015 - The global impact of dementia: an analysis of prevalence, incidence, cost and trends. London: Alzheimer’s Disease International.

[B45] Radford H, Moreno JA, Verity N, Halliday M, Mallucci GR (2015) PERK inhibition prevents tau-mediated neurodegeneration in a mouse model of frontotemporal dementia. Acta Neuropathol 130:633–642. 10.1007/s00401-015-1487-z 26450683 PMC4612323

[B46] Ron D, Habener JF (1992) CHOP, a novel developmentally regulated nuclear protein that dimerizes with transcription factors C/EBP and LAP and functions as a dominant-negative inhibitor of gene transcription. Genes Dev 6:439–453. 1547942 10.1101/gad.6.3.439

[B47] Ron D, Harding HP (2012) Protein-folding homeostasis in the endoplasmic reticulum and nutritional regulation. Cold Spring Harb Perspect Biol 4 10.1101/cshperspect.a013177 PMC3504434 23209157

[B48] Salminen A, Kauppinen A, Suuronen T, Kaarniranta K, Ojala J (2009) ER stress in Alzheimer's disease: a novel neuronal trigger for inflammation and Alzheimer's pathology. J Neuroinflamm 6 10.1186/1742-2094-6-41 PMC2806266 20035627

[B49] Scheper W, Hoozemans JJ (2009) Endoplasmic reticulum protein quality control in neurodegenerative disease: the good, the bad and the therapy. Curr Med Chem 16:615–626. 19199926 10.2174/092986709787458506

[B50] Scheper W, Hoozemans JJ (2015) The unfolded protein response in neurodegenerative diseases: a neuropathological perspective. Acta Neuropathol 130:315–331. 10.1007/s00401-015-1462-8 26210990 PMC4541706

[B51] Schmidt EK, Clavarino G, Ceppi M, Pierre P (2009) SUnSET, a nonradioactive method to monitor protein synthesis. Nat Methods 6:275–277. 10.1038/nmeth.1314 19305406

[B52] Schoonenboom NS, Pijnenburg YA, Mulder C, Rosso SM, Van Elk EJ, Van Kamp GJ, Van Swieten JC, Scheltens P (2004) Amyloid beta(1-42) and phosphorylated tau in CSF as markers for early-onset Alzheimer disease. Neurology 62:1580–1584. 10.1212/01.WNL.0000123249.58898.E0 15136685

[B53] Schröder M, Kaufman RJ (2005) The mammalian unfolded protein response. Annu Rev Biochem 74:739–789. 10.1146/annurev.biochem.73.011303.074134 15952902

[B54] Segev Y, Barrera I, Ounallah-Saad H, Wibrand K, Sporild I, Livne A, Rosenberg T, David O, Mints M, Bramham CR, Rosenblum K (2015) PKR inhibition rescues memory deficit and ATF4 overexpression in ApoE ε4 human replacement mice. J Neurosci 35:12986–12993. 10.1523/JNEUROSCI.5241-14.2015 26400930 PMC6605432

[B55] Sekine Y, Zyryanova A, Crespillo-Casado A, Fischer PM, Harding HP, Ron D (2015) Stress responses. Mutations in a translation initiation factor identify the target of a memory-enhancing compound. Science 348:1027–1030. 10.1126/science.aaa6986 25858979 PMC4538794

[B56] Selkoe DJ (2001) Alzheimer's disease: genes, proteins, and therapy. Physiol Rev 81:741–766. 11274343 10.1152/physrev.2001.81.2.741

[B57] Shankar GM, Li S, Mehta TH, Garcia-Munoz A, Shepardson NE, Smith I, Brett FM, Farrell MA, Rowan MJ, Lemere CA, Regan CM, Walsh DM, Sabatini BL, Selkoe DJ (2008) Amyloid-beta protein dimers isolated directly from Alzheimer's brains impair synaptic plasticity and memory. Nat Med 14:837–842. 10.1038/nm1782 18568035 PMC2772133

[B58] Sidrauski C, Acosta-Alvear D, Khoutorsky A, Vedantham P, Hearn BR, Li H, Gamache K, Gallagher CM, Ang KK, Wilson C, Okreglak V, Ashkenazi A, Hann B, Nader K, Arkin MR, Renslo AR, Sonenberg N, Walter P (2013) Pharmacological brake-release of mRNA translation enhances cognitive memory. Elife 2:e00498. 10.7554/eLife.00498 23741617 PMC3667625

[B59] Sidrauski C, McGeachy AM, Ingolia NT, Walter P (2015a) The small molecule ISRIB reverses the effects of eIF2alpha phosphorylation on translation and stress granule assembly. Elife 4. 10.7554/eLife.05033 PMC4341466 25719440

[B60] Sidrauski C, Tsai JC, Kampmann M, Hearn BR, Vedantham P, Jaishankar P, Sokabe M, Mendez AS, Newton BW, Tang EL, Verschueren E, Johnson JR, Krogan NJ, Fraser CS, Weissman JS, Renslo AR, Walter P (2015b) Pharmacological dimerization and activation of the exchange factor eIF2B antagonizes the integrated stress response. Elife 4:e07314. 25875391 10.7554/eLife.07314 PMC4426669

[B61] Sobów T, Flirski M, Liberski PP (2004) Amyloid-beta and tau proteins as biochemical markers of Alzheimer's disease. Acta Neurobiol Exp (Wars) 64:53–70. 15190680 10.55782/ane-2004-1491

[B62] Spatara ML, Robinson AS (2010) Transgenic mouse and cell culture models demonstrate a lack of mechanistic connection between endoplasmic reticulum stress and tau dysfunction. J Neurosci Res 88:1951–1961. 10.1002/jnr.22359 20143409 PMC4560366

[B63] Tabas I, Ron D (2011) Integrating the mechanisms of apoptosis induced by endoplasmic reticulum stress. Nat Cell Biol 13:184–190. 21364565 10.1038/ncb0311-184 PMC3107571

[B64] Takeuchi H, Iba M, Inoue H, Higuchi M, Takao K, Tsukita K, Karatsu Y, Iwamoto Y, Miyakawa T, Suhara T, Trojanowski JQ, Lee VM, Takahashi R (2011) P301S mutant human tau transgenic mice manifest early symptoms of human tauopathies with dementia and altered sensorimotor gating. PLoS One 6:e21050. 10.1371/journal.pone.0021050 21698260 PMC3115982

[B65] Terry RD, Masliah E, Salmon DP, Butters N, DeTeresa R, Hill R, Hansen LA, Katzman R (1991) Physical basis of cognitive alterations in Alzheimer's disease: synapse loss is the major correlate of cognitive impairment. Ann Neurol 30:572–580. 10.1002/ana.410300410 1789684

[B66] Thomenius MJ, Distelhorst CW (2003) Bcl-2 on the endoplasmic reticulum: protecting the mitochondria from a distance. J Cell Sci 116:4493–4499. 10.1242/jcs.00829 14576343

[B67] Verfaillie T, Garg AD, Agostinis P (2013) Targeting ER stress induced apoptosis and inflammation in cancer. Cancer Lett 332:249–264. 10.1016/j.canlet.2010.07.016 20732741

[B68] Wek RC, Jiang HY, Anthony TG (2006) Coping with stress: eIF2 kinases and translational control. Biochem Soc Trans 34:7–11. 10.1042/BST20060007 16246168

[B69] West MJ, Coleman PD, Flood DG, Troncoso JC (1994) Differences in the pattern of hippocampal neuronal loss in normal ageing and Alzheimer's disease. Lancet 344:769–772. 7916070 10.1016/s0140-6736(94)92338-8

[B70] Yoshiyama Y, Higuchi M, Zhang B, Huang SM, Iwata N, Saido TC, Maeda J, Suhara T, Trojanowski JQ, Lee VM (2007) Synapse loss and microglial activation precede tangles in a P301S tauopathy mouse model. Neuron 53:337–351. 10.1016/j.neuron.2007.01.010 17270732

